# Integration of Functional Imaging, Cytometry, and Unbiased Proteomics Reveals New Features of Endothelial-to-Mesenchymal Transition in Ischemic Mitral Valve Regurgitation in Human Patients

**DOI:** 10.3389/fcvm.2021.688396

**Published:** 2021-08-12

**Authors:** Adrien Lupieri, Yasufumi Nagata, Livia S. A. Passos, Dakota Beker-Greene, Katherine A. Kirkwood, Jill Wylie-Sears, Zahra Alvandi, Hideyuki Higashi, Judy W. Hung, Sasha A. Singh, Joyce Bischoff, Robert A. Levine, Elena Aikawa

**Affiliations:** ^1^Division of Cardiovascular Medicine, Center for Excellence in Vascular Biology and Harvard Medical School, Brigham and Women's Hospital, Boston, MA, United States; ^2^Cardiac Ultrasound Laboratory and Harvard Medical School, Massachusetts General Hospital, Boston, MA, United States; ^3^Department of Population Health Science and Policy, Icahn School of Medicine, International Center for Health Outcomes and Innovation Research, New York, NY, United States; ^4^Vascular Biology Program and Department of Surgery, Boston Children's Hospital and Department of Surgery and Harvard Medical School, Boston, MA, United States; ^5^Division of Cardiovascular Medicine, Center for Interdisciplinary Cardiovascular Sciences and Harvard Medical School, Brigham and Women's Hospital, Boston, MA, United States; ^6^Echocardiography Laboratory, Division of Cardiology and Harvard Medical School, Massachusetts General Hospital, Boston, MA, United States; ^7^Department of Human Pathology, Sechenov First Moscow State Medical University, Moscow, Russia

**Keywords:** ischemic mitral regurgitation, mitral valve, endothelial-to-mesenchymal transition, proteomics, echocardiography, histo-cytometry

## Abstract

**Background:** Following myocardial infarction, mitral regurgitation (MR) is a common complication. Previous animal studies demonstrated the association of endothelial-to-mesenchymal transition (EndMT) with mitral valve (MV) remodeling. Nevertheless, little is known about how MV tissue responds to ischemic heart changes in humans.

**Methods:** MVs were obtained by the Cardiothoracic Surgical Trials Network from 17 patients with ischemic mitral regurgitation (IMR). Echo-doppler imaging assessed MV function at time of resection. Cryosections of MVs were analyzed using a multi-faceted histology and immunofluorescence examination of cell populations. MVs were further analyzed using unbiased label-free proteomics. Echo-Doppler imaging, histo-cytometry measures and proteomic analysis were then integrated.

**Results:** MVs from patients with greater MR exhibited proteomic changes associated with proteolysis-, inflammatory- and oxidative stress-related processes compared to MVs with less MR. Cryosections of MVs from patients with IMR displayed activated valvular interstitial cells (aVICs) and double positive CD31+ αSMA+ cells, a hallmark of EndMT. Univariable and multivariable association with echocardiography measures revealed a positive correlation of MR severity with both cellular and geometric changes (e.g., aVICs, EndMT, leaflet thickness, leaflet tenting). Finally, proteomic changes associated with EndMT showed gene-ontology enrichment in vesicle-, inflammatory- and oxidative stress-related processes. This discovery approach indicated new candidate proteins associated with EndMT regulation in IMR.

**Conclusion:** We describe an atypical cellular composition and distinctive proteome of human MVs from patients with IMR, which highlighted new candidate proteins implicated in EndMT-related processes, associated with maladaptive MV fibrotic remodeling.

## Graphical Abstract

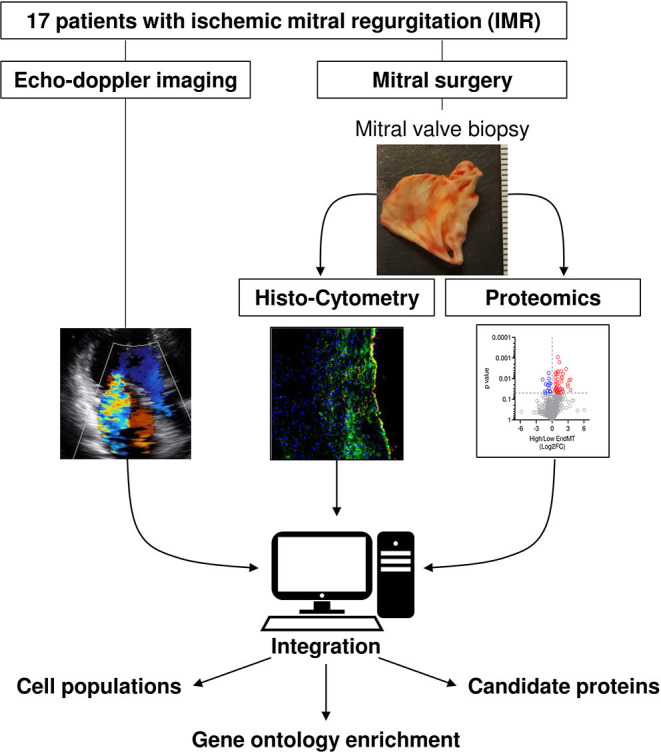

## Introduction

Ischemic mitral regurgitation (IMR) is a common complication after myocardial infarction (MI), and results from modification of the left ventricle (LV) architecture and papillary muscle (PM) displacement. It has been established that IMR is associated with an increase of heart failure and mortality post-MI (1, 2). IMR is a complex disease characterized by systolic blood flow from the LV to the left atrium, resulting from underlying geometric changes, such as limitations of mitral valve (MV) closure by PM tethering and a mismatch between annulus and valve size (3).

Little is known about the biological processes that can lead to adaptation of the MV. Different mechanisms have been proposed; first, that mechanical stress imposed by PM tethering promotes the increase of leaflet area and thickness associated with cellular changes, suggesting an active process of remodeling (4). In addition, it has been suggested that a modulation of leaflet distensibility associates with a change in collagen fiber orientation because of the anisotropic property of collagen fibers, which increases non-aligned fibers in the MV, thereby, increasing extensibility (5–7). However, the role of passive stretching, extracellular matrix (ECM) remodeling, matrix synthesis, and cell growth during MV adaptation remains unclear.

At the cellular level, changes in mitral leaflets during IMR have been associated with endothelial-to-mesenchymal transition (EndMT). During this process, MV endothelial cells (VECs) undergo a transformation of their phenotype toward a mesenchymal-like phenotype, expressing myofibroblastic markers, such as alpha-smooth muscle actin (αSMA) (8, 9). Both transforming growth factor β (TGFβ) and hydrogen peroxide-related oxidative stress primarily contribute to EndMT (10). EndMT within the MV is associated with leaflet extensibility and flexibility leading to MR due to tethering stress that can then be aggravated by MI, as demonstrated in a large animal model (8). Furthermore, angiotensin II pathway inhibition by losartan, which is known to decrease TGFβ signaling, resulted in a significant reduction of leaflet thickness and EndMT, without modification of the adaptive increase of leaflet area (9). Taken together, this suggests a major role of EndMT and TGFβ in the maladaptation of the MV to IMR.

Nevertheless, in human IMR, the effects of ECM remodeling and cellular changes, such as EndMT, are still largely unknown. This gap in knowledge is primarily due to a lack of available human biological material. In this study, we assessed alterations in MV leaflets and performed a comprehensive analysis on a cohort of surgical MV specimens from 17 patients with IMR by corroborating histo-cytometry with functional echocardiographic measurement and unbiased proteomics analyses. Based on this discovery approach, we aimed to bring new insights to the understanding of structural remodeling and cellular changes within the MV.

## Materials and Methods

### Mitral Valves

Adult patients with IMR defined by integrated approach, eligible for MV replacement were enrolled in the National Institutes of Health (NIH) / Canadian Institutes of Health Research (CIHR)-supported Cardiac Thoracic Surgery Network. MV biopsies were collected from subjects undergoing valve replacement, ≥ 1 cm^2^ were excised from the anterior leaflet (A2) region. We excluded any evidence of structural MV disease (chordal or leaflet), ruptured papillary muscle prior MV repair, severe irreversible pulmonary hypertension, contraindication to cardiopulmonary bypass, incapacity to measure the effective regurgitant orifice (ERO) and end-systolic volume index (ESVI), concomitant intra-operative procedure (with the exception of tricuspid valve repair, closure of patent *foramen ovale*, atrial septal defect and Maze procedure), cardiogenic shock, intravenous inotropic treatment, ST segment elevation MI within 7 days, congenital heart disease, evidence of cirrhosis or hepatic synthetic failure, excessive surgical risk, recent history of psychiatric disease, any concurrent disease with life expectance <2 years, and pregnancy at the time of randomization.

### Histopathology

MV pieces from the anterior leaflet (A2) region obtained from 20 patients were snap frozen immediately after excision, then long-term stored at −80°C. Half-part of the frozen MVs were isolated using a razor cut at −20°C for proteomic analysis. The middle portion of frozen MVs were razor cut at −20°C and immediately embedded into Optimum Cutting Temperature compound (OCT, Sakura Finetek, USA). Then, MVs were sectioned into 6 μm slices using a cryostat (Leica CM3050S) followed by histological or immunohistochemical staining.

#### Hematoxylin and Eosin Staining

MV sections were fixed for 20 min in 10% formalin, then stained with successive bath of Harris hematoxylin for 1 min and alcoholic eosin for 1 min. MV thickness was evaluated by the average of 10 measures distributed over the whole leaflet section and analyzed using ImageJ software (NIH).

#### Masson Trichrome Staining

MV sections were fixed 30 min in Bouin solution, then stained with successive baths of Weigert iron hematoxylin for 5 min, Biebrich Scarlet fuchsin for 10 min, phosphomolybdic acid for 30 s, aniline blue for 3 min and acetic acid for 30 s.

#### Picrosirius Red

MV sections were fixed 10 min in 10% formalin, then dipped in a solution of Picric acid 0.1% Sirius red for 3 h, then 1 min in HCl 0.01 N solution. Collagen fibers were observed using polarized light microscope (Nikon), and measure of collagen composition was done from the whole section of MV leaflet by ImageJ software (NIH).

#### Immunohistochemistry

MV cryosections were first fixed 10 min in cold acetone −20°C and secondly fixed for 5 min in 4% paraformaldehyde solution. Sections were incubated for 1 h in blocking solution (PBS, 0.1%; Tween 20, 3% serum) at room temperature. We then incubated primary antibodies (see table below) overnight at 4°C. After 3 repeats of a 5 min wash in PBS, 0.1% Tween 20, sections were incubated with fluorescent-conjugated secondary antibodies (see Table below) for 2 h at room temperature. Slides were washed 3 times for 5 min in PBS containing 0.1% Tween 20 and nuclei were stained with DAPI (4,6-diamidino-2-phenylindole, R37606, Invitrogen), cover slipped, and examined with a Nikon Eclipse Confocal microscope (Nikon, USA).

**Table:** 

**Primary Ab**	**Reference**	**Dilution**	**Secondary Ab**	**Reference**	**Dilution**
Anti-CD31	ab28364	1/50	Anti-rabbit IgG AF647	A21245	1/500
Anti-CD45	MA5-17687	1/500	Anti-rat IgG AF594	A11007	1/500
Anti-αSMA	M0851	1/500	Anti-mouse IgG AF488	A11001	1/500

#### Cell Population Analysis

Confocal microscopy images were analyzed by ImageJ software (NIH). Two methods of measure were performed using the average of 3 independent high power fields (640 × 640 μm) per sample. First, we performed unbiased assessment of the positive area for each staining: CD31+; CD45+ and αSMA+. We then measured the colocalization area for each pair of staining: CD31+ CD45+; CD31+ αSMA+ and CD45+ αSMA+. The single positive areas were calculated by subtraction of double positive areas from the total: CD31+ = (total CD31+)-[(CD31+; CD45+) + (CD31+ αSMA+)]; αSMA+ =(total αSMA+)-[(CD31+ αSMA+) + (CD45+ αSMA+)]; CD45+ = (total CD45+)-[(CD31+ CD45+) + (CD45+ αSMA+)]. The second method was a manual counting of nuclei (DAPI staining) colocalized with single or double positive staining determined by the first analysis, enabling to exclude the non-cellular staining.

### Echocardiography

#### Comprehensive Two-Dimensional Transthoracic Echocardiography

Comprehensive two-dimensional transthoracic echocardiography was performed before the surgical procedure including MV replacement with MV excision with or without coronary-artery bypass grafting. Basic echocardiographic parameters were measured in the core laboratory (Massachusetts General Hospital, Boston, Massachusetts). MV-specific parameters were additionally measured blinded to any clinical and biological information. Left ventricular end-diastolic/end-systolic volumes and ejection fraction were measured by the biplane method of disks summation. The volumes were indexed by body surface area. *Vena contracta* width (VC) was measured to quantify MR severity (11). MV-specific parameters, open anterior and posterior leaflet lengths were measured from the leaflet insertion to the tip in the apical three chambers view during diastasis. Leaflet thickness was measured in the middle portion of the leaflet in the parasternal long-axis view to minimize the impact of stretch. Closure leaflet length was measured as the tissue length between anterior and posterior annuluses and the tenting area was measured as the area surrounded by leaflets and the line between annuluses on apical 3 chambers-view in mid-systole. The patients were categorized into two groups according to quantitative MR severity with VC to eliminate assumption error and hemodynamic factors in MR classification, patients displaying VC ≦ 7 mm were defined as moderately severe MR (MSMR), patients displaying VC > 7 mm were classified as severe MR (SMR).

### Proteomics

#### Label-Free Proteomics Preparation for Human MV Samples

A total of 27 samples were processed together for proteomics analysis. Out of those, 7 samples were not included in the final proteomic analysis due to the unavailability of the echocardiography measures and the tissue cryosections. Each piece of frozen MV was pulverized in liquid nitrogen, then sonicated in RIPA buffer (Pierce, 89900) complemented with 1% protease inhibitor cocktail. Protein precipitation was performed with chloroform:methanol (2:1) method and redissolve using a solution of urea 6M/thiourea 2M/TEAB 100 mM (pH 8). 15μg of protein were cleaved using trypsin/LysC cocktail at 37°C overnight. The peptides were desalted using Oasis HLB column (Waters, USA) and dried with a Speed Vacuum concentrator (SPD1010, Thermo Fisher Scientific, USA). After re-suspension in 40 μl of 5% mass spectrometry grade acetonitrile (Thermo Fisher Scientific, USA) and 5% formic acid (Sigma-Aldrich, USA).

#### Data-Dependent Acquisition

The peptides were analyzed using the Orbitrap Fusion Lumos Tribrid mass spectrometer (Thermo Fisher Scientific) fronted with an Easy-Spray ion source and coupled to an Easy-nLC1000 HPLC pump (Thermo Fisher Scientific). The peptides were separated using a dual column set-up: An Acclaim PepMap RSLC C18 trap column, 75 μm X 20 mm; and an EASY-Spray LC heated (45°C) column, 75 μm × 250 mm (Thermo Fisher Scientific). The gradient flow rate was 300 nl/min from 5 to 21% solvent B (acetonitrile/0.1% formic acid) for 75 min, 21 to 30 % Solvent B for 15 min, followed by 10 min of a ‘jigsaw wash’, alternating between 5 and 95 % Solvent B. Solvent A was 0.1% formic acid. The instrument was set to 120 K resolution, and the top N precursor ions in a 3 second cycle time (within a scan range of 375–1,500 m/z; isolation window, 1.6 m/z; ion trap scan rate, normal) were subjected to collision induced dissociation (collision energy 30%) for peptide sequencing (or MS/MS). Dynamic exclusion was enabled (60 s).

#### Mass Spectrometric Data Analysis

The MS/MS spectra were queried against the human UniProt database (downloaded on August 1, 2018; 155, 133 sequencing) using the HT-SEQUEST search algorithm, *via* the Proteome Discoverer (PD) Package (version 2.2, Thermo Fisher Scientific). Methionine oxidation and n-terminal acetylation were set as a variable modification, and carbamidomethylation of cysteine was set as a fixed modification. The enzyme was set to trypsin (full), with a maximum of four missed cleavages, using a 10 ppm precursor tolerance window and a 0.02 Da fragment tolerance window. Peptides were filtered based on a 1% FDR based on the reverse database (decoy) results (12, 13). In order to quantify peptide precursors detected in the MS1 but not sequenced from sample to sample, we enabled the “Feature Mapper” node. Chromatographic alignment was done with a maximum retention time (RT) shift of 10 min and a mass tolerance of 10 ppm. Feature linking and mapping settings were, RT tolerance minimum of 0 min, mass tolerance of 10 ppm and signal-to-noise minimum of five. Precursor peptide abundances were based on their chromatographic intensities and total peptide amount was used for normalization. Peptides assigned to a given protein group, and not present in any other protein group, were considered as unique. Consequently, each protein group is represented by a single master protein (PD Grouping feature). We used unique and razor peptides per protein for quantification and filtered for proteins with two or more unique peptides.

The mass spectrometry proteomics data have been deposited to the ProteomeXchange Consortium via the PRIDE (14) partner repository with the dataset identifier PXD025096 and 10.6019/PXD025096.

### Statistical Analysis

Non-parametric unpaired *T*-test were calculated using Excel to compare the protein abundance between subgroups of patients.

Simple linear correlation from proteomics data were calculated with Excel functions. For each protein we determined the correlation R = CORREL([array 1], [array 2]); T statistic was calculated by T = ([R]^*^SQRT([number of pairs of data]-2)/SQRT(1-[R]^∧^2)); *p*-value was determined by *p* = TDIST([T], [degrees of freedom], [number of tails]).

Simple linear regression using echocardiography, histology or cytometry parameters were calculated using GraphPad Prism 8.4 software.

Gene ontology enrichment were identified using g:Profiler Homo sapiens database (https://biit.cs.ut.ee/gprofiler) using g:GOSt (15). Functional enrichment was assessed from ordered list of protein (ascending *p*-value). Benjamini-Hochberg False Discovery Rate correction was applied to calculate the adjusted *p*-value.

Univariable linear regression models were used to explore the relationships between echocardiographic measures of mitral valve disease including mid-systole tenting area, *vena contracta* (VC), clinical characteristics including age and sex, and histo-cytometry measurements of CD45+/CD31+/αSMA+ immunostainings from excised mitral valve tissue samples. Logarithmic transformations were used as appropriate for non-normally distributed continuous variables. Variables showing a nominal association with the independent variable (α = 0.10) were considered for inclusion in a multivariable model of VC, chosen as the echocardiographic measure most representative of mitral regurgitation. For all models, statistical significance was considered at the α = 0.05 level; as these were hypothesis-generating analyses, no adjustment was made for multiple comparisons. All these analyses were conducted using SAS v9.4.

## Results

### Echocardiography Analysis

**Figure 1:**
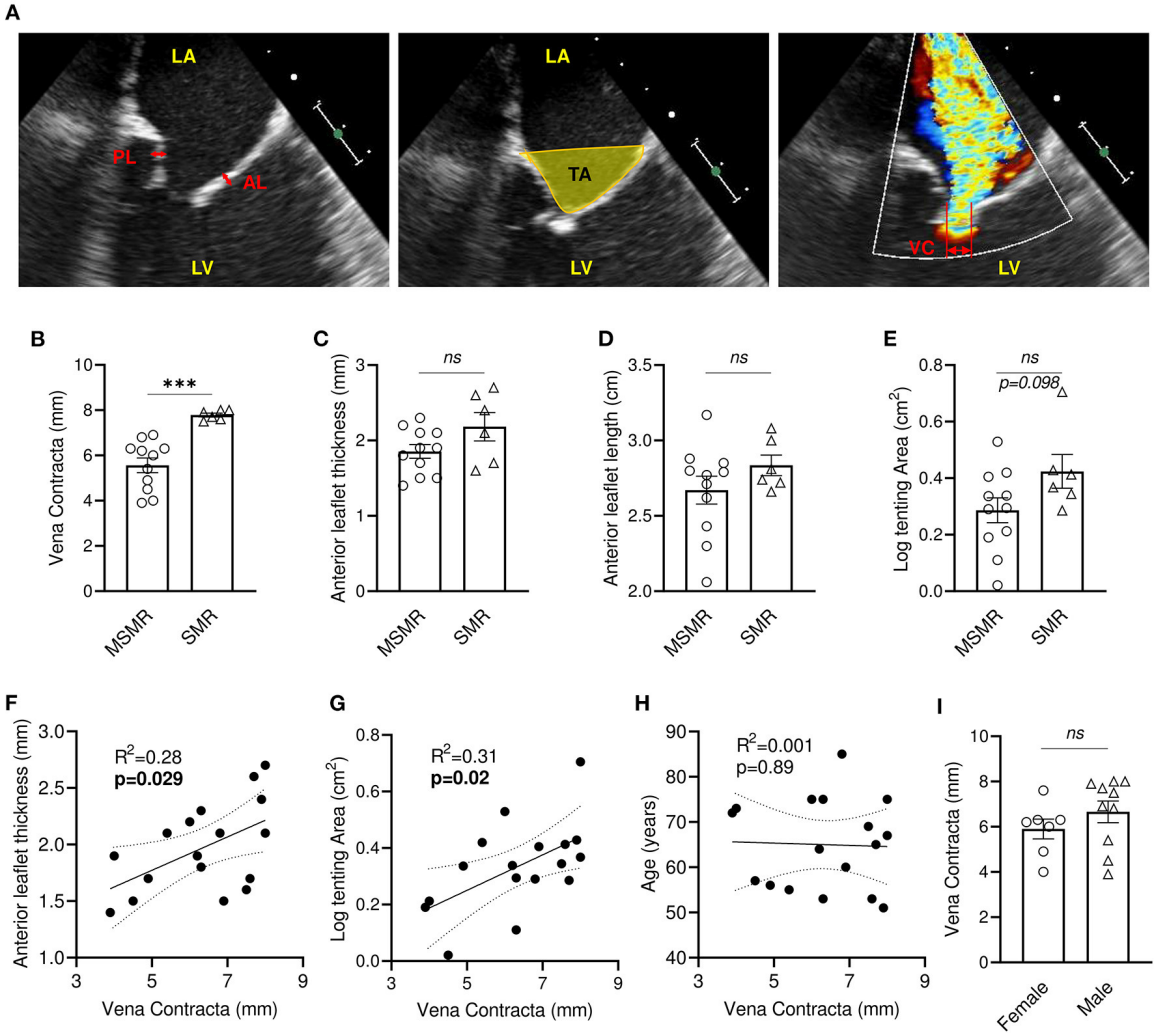
Mitral regurgitation is associated with leaflet thickening and increased tenting area in patients with IMR. **(A)** Anterior and posterior mitral valve leaflet thickness were measured in the middle portion of the leaflet (left: bidirectional red arrows). Tenting area was measured as the area surrounded by leaflets and the line between annuluses on apical 3ch-view in mid-systole (middle: yellow area). Vena contracta (VC) width was measured as the narrowest width of mitral regurgitation jet (right: bidirectional yellow arrow). Comparison of VC **(B)**, anterior leaflet thickness **(C)**, anterior leaflet length **(D)**, tenting area **(E)** between patients presenting moderately severe MR (MSMR) (*n* = 11) and severe MR (SMR) (*n* = 6). Linear correlation between VC and anterior leaflet thickness **(F)**, tenting area **(G)**, anterior leaflet length **(D)** and age **(H)**. **(I)** VC in female (*n* = 7) and male (*n* = 10). LA, left atrium; LV, left ventricle; AL, anterior mitral valve leaflet; PL, posterior mitral valve leaflet. ns, no-significant, ***p < 0.001. Thin dotted line shows the 95% confidence interval.

Two-dimensional transthoracic echocardiography was performed before the surgical procedure to evaluate MV function (Figure 1A). Echocardiographic evaluation of mitral regurgitation has been assessed by *vena contracta* (VC) measurement. All patients displayed a VC ≥ 4 mm (4–8 mm, *n* = 17), among which 11 patients presented moderately severe MR (MSMR) (VC between 4 and 7 mm) (16) and six patients exhibited severe MR (SMR) (VC > 7 mm) (16) (Figure 1B). Patients with MSMR or SMR did not present significant variation of anterior mitral leaflet (AL) thickness and length (Figures 1C,D). Nevertheless, simple linear regression model showed a significant positive correlation between AL thickness and VC (*R*^2^ = 0.28, *p* = 0.029) (Figure 1F). Measurement of the MV tenting area at mid-systole displayed a non-normal distribution (Shapiro-Wilk test, *p* = 0.012), which required a logarithmic transformation. Patients with SMR tends to have large tenting area than those with MSMR (*p* = 0.098) (Figure 1E), and there was a significant positive correlation between VC and the tenting area (*R*^2^ = 0.31, *p* = 0.019) (Figure 1G). Conversely, the linear regression model did not show an association of VC with anterior leaflet length (*R*^2^ = 0.002, *p* = 0.86) (Supplementary Figure 1A). VC, AL thickness, and tenting area showed large heterogeneity (Supplementary Table 1), suggesting that in our cohort, tethering stress and left ventricle architecture modification had differing effects after MI. In our cohort, VC is not significantly associated with the age of the patient (*R*^2^ = 0.001, *p* = 0.89) and does not vary significantly by gender (*p* = 0.184) (Figures 1H,I).

### Histological Analysis

All samples used in this study were obtained from patients undergoing surgical excision of the anterior MV after IMR. Procedures were performed and registered with the Cardiothoracic Surgical Trial Network (CTSN). We obtained 20 tissue specimens from which two were excluded based on the histological appearance (samples designated Ex1, Ex2), due to their predominant myocardial composition, as presented in Supplementary Figure 2A. An additional sample was excluded based on the mean protein abundance, as determined by proteomics criteria (Ex1, Ex2, Ex3, in Supplementary Figure 2B).

**Figure 2:**
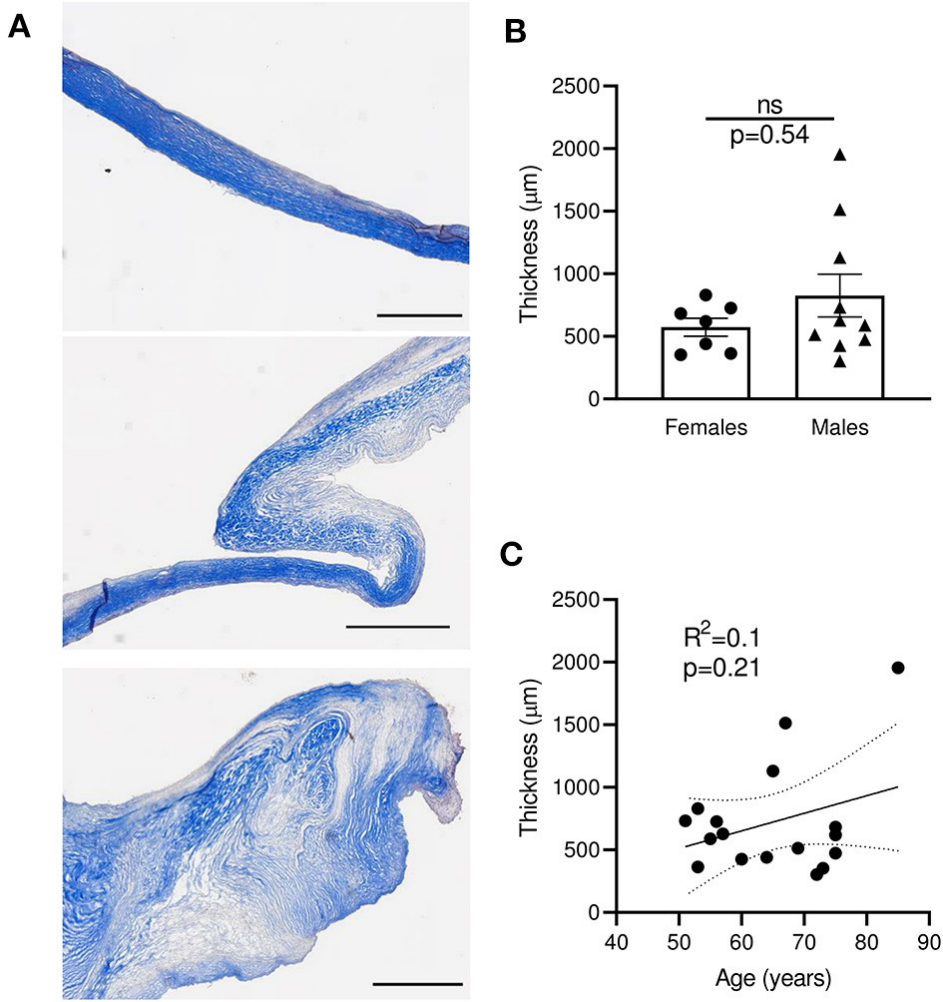
Sections of human MV with MR display large heterogeneity of thickness, independently of age and gender. **(A)** Histological sections of human mitral valves representative of three different grades of histological remodeling (patients #7, #4, #11, Supplementary Table 2), stained with Masson trichrome (scale bar represents 1 mm). **(B)** Thickness of MV leaflet section from male vs. female samples. **(C)** Correlation between the age of donor and the MV thickness for each donor. Thin dotted line shows the 95% confidence interval. ns, no significant.

Masson's trichrome staining of the mitral leaflet sections showed a large heterogeneity in size, morphology, and configuration of the different layers (Figure 2A and Supplementary Table 2). We observed three major groups of histological patterns: thin leaflets with high fibrotic content (sample #7, Figure 2A top), an intermediate phenotype with organized layers (sample #4, Figure 2A middle), and samples with largely disorganized structures and heterogeneous fibrotic composition (sample #11, Figure 2A bottom). The observed thickness variability was not related to gender (*n* = 7–10; *p* = 0.54) (Figure 2B), and was not correlated with the age of patients at time of surgery (*R*^2^ = 0.1, *p* = 0.21) (Figure 2C). These results suggest that different processes, independently of gender and age, are linked to MV post-MI adaptation, leading to IMR.

### Echocardiography/Proteomic Integration

**Figure 3:**
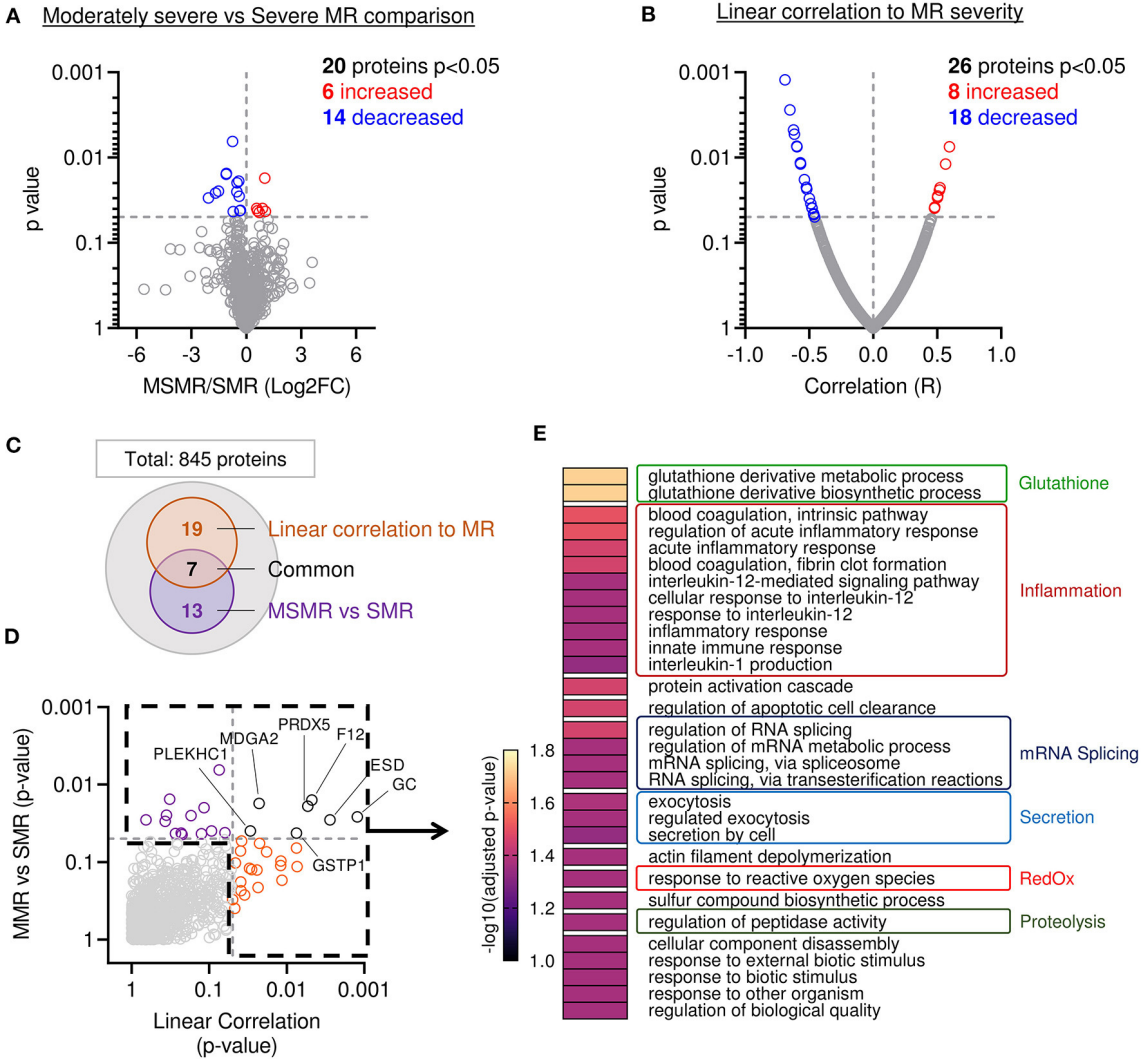
Mitral leaflets from patients with higher MR display proteomic change associated to proteolysis-, inflammatory-, and oxidative stress-related processes. **(A)** Comparison of proteome composition from the samples having moderately severe MR (MSMR *n* = 1) vs. the samples with severe MR (SMR *n* = 6) Volcano plot displaying the log2 fold-change (x-axis) against the statistical *p*-value (log scale) for all 845 proteins detected. Proteins with significantly increased expression (Log2FC > 0, *p* < 0.05) in SMR group are shown in red, while the proteins with significantly decreased expression (Log2FC > 0, *p* < 0.05) are expressed in blue. **(B)** Comparison of proteomic composition using linear correlation with MR level quantification (vena contracta). Dot plot displaying the R (x-axis) against the statistical *p*-value (log scale) for all 845 patients detected. Proteins with the significant and positive correlation (R > 0, *p* < 0.05) to MR are shown in red, while the proteins with significant and negative correlation (R < 0, *p* < 0.05) are presented in blue. **(C)** Venn diagram displaying modulated proteins obtain from the linear correlation to MR (orange) and MSMR vs. SMR comparison (purple), common proteins were identified in black. **(D)** Dot plot exhibiting *p*-value 845 patients identified from both analyses presented linear correlation to MR (orange), MSMR vs. MR comparison (purple), common proteins (black). **(E)** Heatmap of gene ontology biological process (GO:BP) enriched from protein significantly modulated.

Proteome composition of the MVs from 11 patients displaying MSMR (VC ≦ 7 mm) was compared to the MV's proteome from the six patients displaying SMR (VC > 7 mm). From the 845 proteins identified in these samples, 20 showed a significant difference in protein abundance (6 increased and 14 decreased) (Figure 3A). To corroborate these results, we performed a second analysis using a linear regression model between the abundance of each protein and the MR severity of each patient, as assessed by echo-doppler measurement of the VC. From the 845 proteins identified in our sample group, 26 showed a significant correlation to protein abundance (8 increased and 18 decreased) (Figure 3B). A total of 39 proteins were found related to MR (Figures 3C,D and Supplementary Table 3), with 7 proteins significantly changed in both approaches (Figures 3C,D; black).

Next, we compared the main biological processes enriched in the group of significantly modulated proteins from both methods: comparison of MSMR vs. SMR groups and linear correlation to MR (Figures 3C,D). The analysis of biological process enrichment revealed an extensive implication of glutathione derivative metabolism (GO:1901685, GO:1901687) (Figure 3E), related to ESD (Esterase D) and GSTP1 (Glutathione S-Transferase Pi 1) decreased with the increase of MR. We also observed involvement of several inflammatory processes, such as regulation of the acute inflammatory response (GO:0002673, GO:0002526), blood coagulation (GO:0007597, GO:0072378), innate immune response (GO:0045087), and response to interleukin-12 (GO:0035722, GO:0071349, GO:0070671) and−1 (GO:0032612) (Figure 3E). 11 proteins matched with inflammatory-related GO:BP, including A2M (Alpha-2-Macroglobulin), C3 (Complement C3) were increased in samples with more severe MR, which were opposite to CAPZA1 (CapZ Alpha-1), CFL1 (Cofilin 1), CD44, F12 (Coagulation Factor XII), GSTP1, HMGB1 (High Mobility Group Box 1), PRDX5 (Peroxiredoxin 5), PSME1 (Proteasome Activator Subunit 1), S100A13 (S100 Calcium Binding Protein A13), which were decreased. We also found an enrichment of response to reactive oxygen species (GO:0000302) (Figure 3E), linked to GSTP1 and PRDX5, both decreased in samples with higher MR. Additionally, we observed an enrichment of the proteolysis-related process named regulation of peptidase activity (GO:0052547) (Figure 3E) connected to the reduction of CD44, HMGB1, PSME1, RCN3 (Reticulocalbin 3), and the induction of TIMP3 (Tissue Inhibitor of Metalloproteinases 3) in MVs with superior MR.

### MV Cell Composition

**Figure 4:**
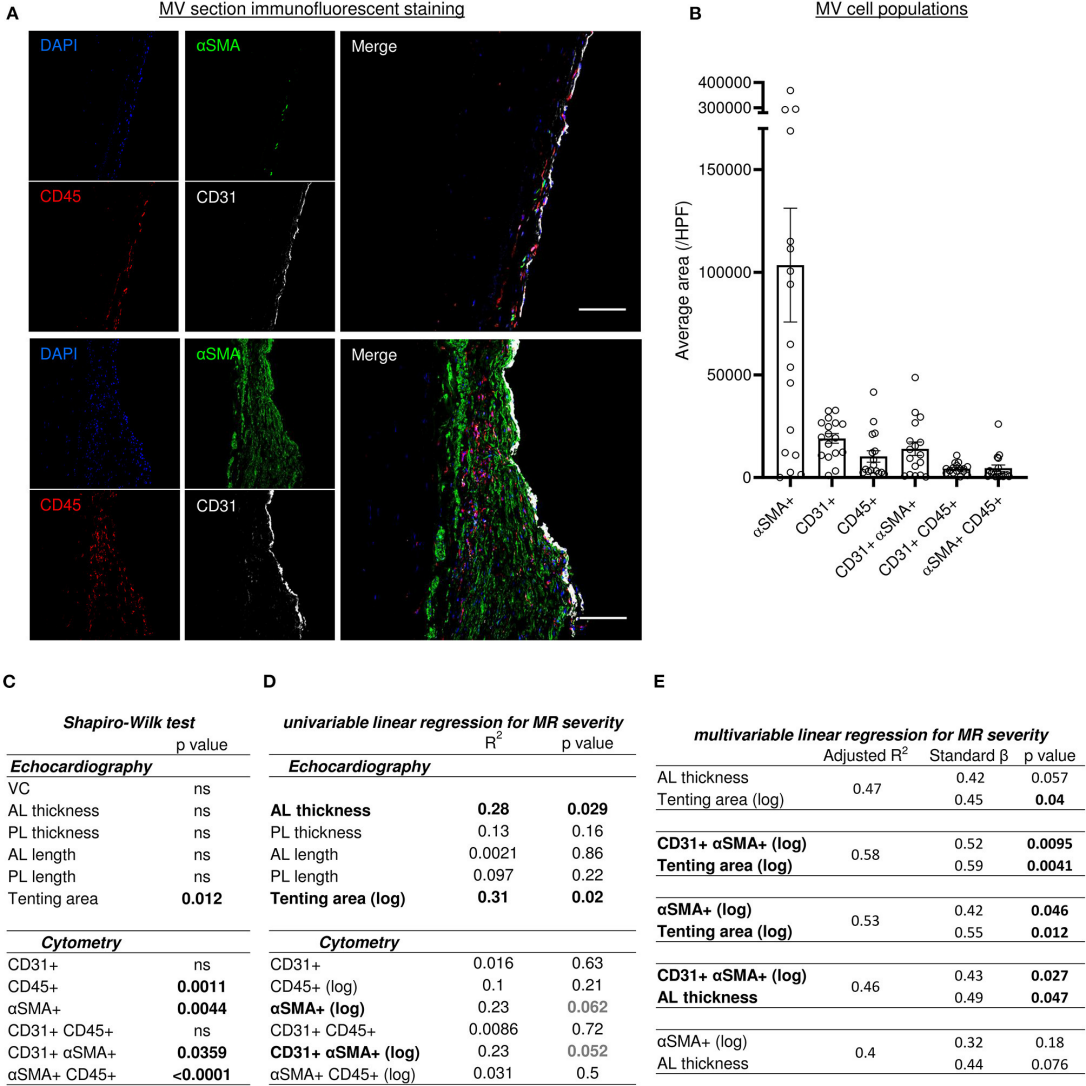
Histo-cytometry assessment of MV cell composition correlated with echocardiography measures revealed that MR is concomitantly associated to EndMT and leaflet's thickness or tenting. **(A)** Representative MV section from patient with different range of cellular composition, (patients #14 upper and #12 bottom) stained with immunofluorescent co-staining of DAPI (blue), αSMA (green), CD45 (red), and CD31 (white), merge on the left panel (scale bar represents 100 μm). **(B)** Cell count per high power field (HPF) for αSMA single positive cells (αSMA+), CD45 single positive cells (CD45+), CD31 single positive cells (CD31+), along with CD31/αSMA double positive (CD31+ αSMA+) and CD31/CD45 double positive (CD31+ CD45+). **(C)** Shapiro-Wilk test of data distribution. **(D)** Univariable linear regression with VC. **(E)** Multivariable linear regression with VC. MV, mitral valves; VC, vena contracta; PL, posterior mitral leaflet; AL, anterior mitral leaflet; SP, single positive.

The quadruple immunofluorescent staining (CD31, αSMA, CD45 and DAPI) associated with sub-analysis of single positive and double positive areas identified different cell populations within the MV leaflet is presented in Figures 4A,B. Average areas of each staining are included in Figure 4B and quantification by counting of each cell type is presented in Supplementary Figure 3A.

We assessed an αSMA as a marker of activated valvular interstitial cells (aVICs), also known as valvular myofibroblast-like cells. αSMA single positive (αSMA+) cells were heterogeneously present in the different MV donors and showed a strong negative correlation with collagen content detected by picrosirius red staining (*R*^2^ = 0.37, *p* = 0.009) (Supplementary Figure 3B), but did not significantly correlate with leaflet thickness (*R*^2^ = 0.186, *p* = 0.08) (Supplementary Figure 3C).

The population of CD45 single positive cells (CD45+) indicates leukocytes infiltrated the MV. We observed low levels of leukocyte infiltration (<10 CD45+ cells per high power field [HPF]) except in four samples (#3, #5, #12 and #17), which displayed >18 CD45+ cells per HPF (35.3; 20; 18.7; 21.3, respectively) (Supplementary Figure 3A). Furthermore, there was a significant negative correlation between CD45+ cells and collagen content (*R*^2^ = 0.42, *p* = 0.0046) (Supplementary Figure 3D), but not with leaflet thickness (Supplementary Figure 3E). On the other hand, the number of leukocytes (CD45+) and aVIC (αSMA+) was correlated significantly (*R*^2^ = 0.26, *p* = 0.037) (Supplementary Figure 3F).

Endothelial cell population is characterized by CD31 single positive (CD31+) staining and the number of this cell type was consistent in all samples (Figure 4B), and associated with the endothelial layer surface measurements per field. These results suggest that endothelial cell content is not altered during post-MI IMR progression. However, we identified two additional CD31+ populations, including double positive CD31+ αSMA+ and CD31+ CD45+ cells. The CD31+ αSMA+ subpopulation demonstrated a wide variability between MVs (Figures 4A,B and Supplementary Figure 3A).

**Figure 5:**
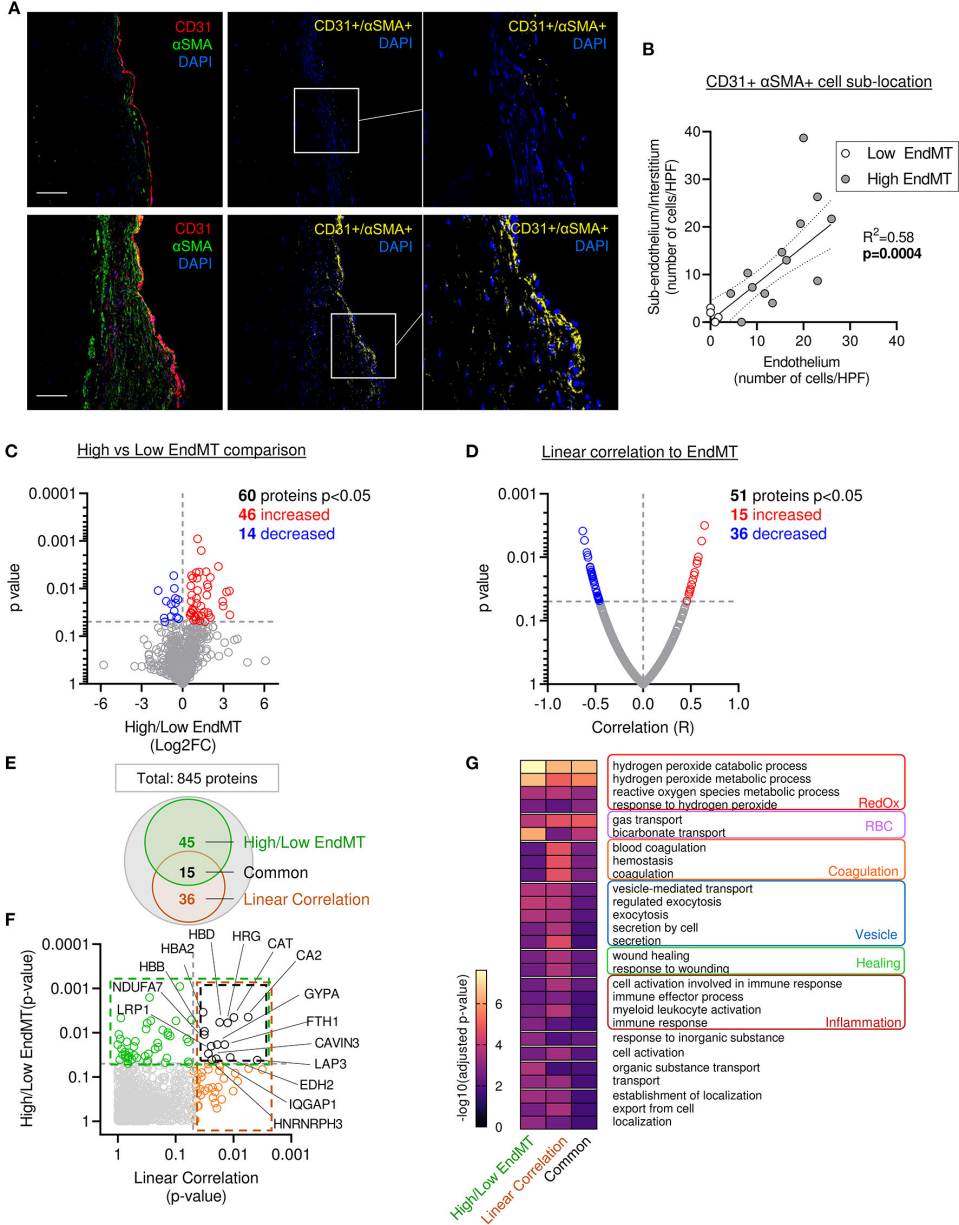
Mitral leaflets displaying EndMT are associated with proteomic change associated to, oxidative stress-, inflammatory- and vesicle-related processes. **(A)** Representative mitral valve section from patient with different range of cellular composition, (patients #10 upper panel and #12 bottom panel) stained for DAPI (blue), CD31 (red), αSMA (green) merge on the left panel (scale bar represent 100 μm). CD31 and αSMA double positive areas are represented in yellow (two right panels). **(B)** Sub localization of EndMT cells (CD 31+αSMA + DAPI+) between endothelium layer (x axis) and sub endothelium and interstitium area (y axis). **(C)** Comparison of proteomic composition from the four samples having low level of EndMT (white dots in **B**) vs. the 13 other samples with higher level of EndMT (gray dots in **B**). Volcano plot displaying the Log2 fold change (x axis) against the statistical *p*-value (log scale) for all 845 proteins detected. Proteins with significantly increased expression (Log2FC > 0, *p* < 0.05) in sample with high level of EndMT are shown in red, while the proteins with significantly decreased expression (Log2FC > 0, *p* < 0.05) are presented in blue. **(D)** Comparison of proteomic using linear correlation with EndMT quantification (CD31+αSMA+ area normalized to total CD31+). Dot plot displaying *R*^2^ (x axis) against the statistical *p*-value (Log scale) for all 845 patients detected. Proteins with significant and positive correlation (*R* > 0, *p* < 0.05) to EndMT are shown in red, while the proteins with significant and negative correlation (*R* > 0, *p* < 0.05) are presented in blue. **(E)** Venn diagram displaying the significantly modulated proteins obtain from the linear correlation to EndMT (dark orange) and Low vs. High EndMT comparison (green) and common proteins (black). **(F)** Dot plot exhibiting *p*-value of 845 patients identified from both analyses. Common proteins are indicated in black. **(G)** Gene ontology biological process (GO:BP) from protein significantly modulated in the low vs. high EndMT comparison, linear correlation to EndMT, or only in the common proteins.

Previous preclinical studies in a large animal model reported the presence of endothelial cells undergoing EndMT, characterized by the co-expression of endothelial markers (e.g., CD31, VE-Cadherin, NOS 3) and mesenchymal markers (e.g., αSMA, calponin, SM22a, versican) (9, 10, 17). Similarly, we observed the presence of cells co-expressing CD31 and αSMA (CD31+ αSMA+) in the MVs from patients with severe IMR. Quantitative analysis of confocal fluorescent microscopy images applying two complementary methods (Supplementary Figures 4A,B), demonstrated significantly correlated values (*R*^2^ = 0.463, *p* = 0.0026) (Supplementary Figure 4A). The assessment of EndMT cells' sub-localization within the leaflet layer showed CD31+ αSMA+ DAPI+ cells in the endothelial layer, as well as in the sub-endothelium and in valve interstitium (Figure 5A). The number of cells in each compartment was positively correlated (*R*^2^ = 0.58, *p* = 0.0004), supporting the hypothesis of cell migration from the endothelium to the interstitium during EndMT (Figure 5B).

### Echocardiography/Histo-Cytometry Integration

Various models of linear regression were applied to study the possible relationship between cell populations observed within the MV and clinical parameters measured by echocardiography. The variables with non-normal distribution including tenting area, CD45+, αSMA+, CD31+αSMA+ and αSMA+CD45+ cells (Figure 4C), were then transformed by logarithmic function for univariable and multivariable linear regression analysis (Figures 4D,E). *Vena contracta* (VC) was used as a numeral parameter representative of MR severity. Univariable and multivariable regression models were used to assess the relationship between clinical echocardiographic parameters measured at the time of surgery and histo-cytology parameters determined from tissue samples.

Univariable linear regression to MR with other echocardiography measurements demonstrated a significant association with anterior leaflet (AL) thickening and tenting area (*R*^2^ = 0.28, *p* = 0.029 and *R*^2^ = 0.31, *p* = 0.02, respectively) but no other echocardiography measurements (Figure 4D). Univariable linear regression demonstrated a trend toward an association between MR and cytometry measurements, including aVICs (αSMA+) (*R*^2^ = 0.23, *p* = 0.062) and cells undergoing EndMT (CD31+αSMA+) (*R*^2^ = 0.23, *p* = 0.052) (Figure 4D). Multivariable linear regression analysis was used to test which combination of parameter was the best model to predict MR severity by including variables selected in univariable analyses. Two variables were included in models according to the limited number of our cohort. Multivariable linear regression modeling MR dependent variables did not show a significant concomitant association with AL thickness and tenting area (Figure 4E); even though the relationship was close to significant (*R*^2^ = 0.47, AL thickness *p* = 0.057, tenting area *p* = 0.04). However, when utilizing a multivariable linear regression model that incorporates MR dependent variables, we observed that MV tenting area and aVICs (αSMA+) (*R*^2^ = 0.53), as well as tenting area and cells undergoing EndMT (CD31+αSMA+) (*R*^2^ = 0.58), are significantly associated with MR severity (Figure 4E). Additionally, a multivariable linear regression model for MR severity displayed association with AL thickness and EndMT cells (CD31+αSMA+), but not with AL thickness and aVICs (αSMA+) (Figure 4E). These findings suggest MR severity is determined by the combination of geometric and histopathologic changes.

### Histo-Cytometry/Proteomic Integration

The four samples displaying lower EndMT rate (Supplementary Figure 4A; white dots) were used as an internal control to investigate the proteomic changes specifically related to the EndMT process in the MV. From the 845 proteins identified in our samples, 60 showed a significant difference in protein abundance (46 increased and 14 decreased) (Figure 5C and Supplementary Table 4).

To support these results, we performed a second analysis using a linear regression model that considered the abundance of each protein and the EndMT level from each sample. For this, we used an unbiased measure of CD31+ αSMA+ area normalized as percent of the total CD31+ area. From the 845 proteins identified in our sample group, 51 showed a significant difference in protein abundance (15 increased and 36 decreased) (Figure 5D and Supplementary Table 4). Among them, 15 proteins were found significantly changed in both analyses (Figures 5E,F and Supplementary Table 4).

Furthermore, we found a significant difference in the abundance of proteins previously reported to be involved in the EndMT process (COL1A, CAST, TAGLN2, CAT, APOA1, BLVRB), or described in a comparable biological process of epithelial-to-mesenchymal transition (EMT) (HRG, EHD2, FGL2, FBN1, CA2, LASP1, HMGB1, LMCD1, NID1, FTH1, TGFB1I1, PRDX2, SRPX, LRP1, PLA2G2A, MAOA, TTN, IQGAP1) (Supplementary Table 5), which enhanced the confidence in our hypothesis that EndMT is highly involved in MV pathology. Moreover, 73 other proteins that were significantly altered, but have not been associated with EMT or EndMT, comprise new candidate proteins that might have key functional roles or work as biomarkers (Supplementary Table 4).

Next, we compared the main GO:BP enriched in the list of significantly modulated proteins from the various methods of analysis: comparison of high vs. low EndMT groups, the EndMT linear correlation, as well as the proteins common to both analyses (Figures 5E–G and Supplementary Table 4). The analysis focused on the 50 more significant GO:BP and selected the ones shared in the 3 approaches of analysis (Figure 5G).

The analysis of biological process enrichment revealed an extensive effect of several oxidative stress-related processes enriched in MV displaying high EndMT, including hydrogen peroxide metabolic processes (GO:0042744, GO:0042743), response to hydrogen peroxide (GO:0042542) and reactive oxygen species metabolic process (GO:0072593) (Figure 5G) subsequently to the upregulation of CAT, HBA2, HBB, HBD, PRDX2 and SNCA, as well as the downregulation of ARF4 and BST1 (Supplementary Table 4). CAT has been previously implicated in EndMT, PRDX2 has shown involvement in EMT (Supplementary Table 5), whereas SNCA and erythrocyte-associated proteins HBA2, HBB, HBD have not been linked to either processes.

In addition, we observed an involvement of inflammatory processes, which included cell activation in the immune response (GO:0002263), immune response (GO:0006955), immune effector process (GO:0002252) and myeloid leukocyte activation (GO:0002274) (Figure 5G). Proteins associated with these inflammatory processes revealed proteins previously associated with EndMT, including APOA1, CAT, COL1A1, as well as proteins previously connected to EMT, including FGL2, FTH1, HMGB1, HRG, IQGAP1, LRP1 and PLA2G2A. However, 15 of them have never been related to EMT or EndMT, including APCS, BST1, FTL, HBB, HSPD1, JCHAIN, PPBP, SERPINB6, and SNCA (Supplementary Tables 4, 5) suggesting potentially new players in this biological process.

Assessment of gene ontology enrichment also revealed the association of vesicle-mediated processes, such as vesicle-mediated transport (GO:0016192), regulated exocytosis (GO:0045055), endocytosis (GO:0006897), secretion (GO:0046903), and secretion by cell (GO:0032940) (Figure 5G). Among proteins connected to these biological processes, 3 have been related to EndMT (APOA1, CAT, TAGLN2), 7 have been associated to EMT (EHD2, FGL2, FTH1, HMGB1, HRG, TTN) (Supplementary Table 5), while 12 of them have never been linked to either process (ARF4, BST1, ERP70, F13A1, FTL, HBA2, HBB, JCHAIN, PPBP, SCARB2, SERPINB6, SNCA, SPTB) (Supplementary Table 4), indicating potential new members of this biological function.

## Discussion

In this study, we used MVs from patients with IMR to explore histologic and cellular characteristics of the MV leaflet, which were then corroborated with functional echocardiographic measurements and proteome composition. Our results indicate a multifactorial disease mediated by alterations in MV thickening and architecture related to changes in the cellular composition, such as EndMT and VIC activation.

In our cohort of patients displaying IMR, all *vena contracta* measurements were ≥ 4 mm (average 6.34 ± 1.39 mm), confirming diagnosis of severe MR (16). We observed a large discrepancy in leaflet thickness (from 1.4 to 2.7 mm based on echo-guided measurements), which revealed a variety of responses to IMR. The standard value for both anterior and posterior leaflet thickness established via echocardiography measurement was approximately 1.6 mm (4), indicating that not all patients with IMR displayed thickening of their mitral leaflets. However, we observed that anterior leaflet thickness is positively and significantly correlated with the regurgitation severity, confirming previous observations that leaflets tend to thicken following MR (18). Conversely, severity of MR was not correlated to mitral leaflet length variation.

Mitral tenting is the geometric feature characterized by insufficient systolic leaflet displacement toward the annulus with restricting leaflet coaptation, which is reflected by increased tethering forces on the MV and reduced closing forces. Tethering forces are increased by regional and global LV dilatating remodeling through chordae and papillary muscles with increased tenting area, resulting in MR deterioration (3, 19). We observed in our cohort a positive linear association between MR severity measured as *vena contracta* and tenting area (Figure 1G), which is consistent with previous reports demonstrating an important relationship between the MR severity in patients and morphological changes in the coordination of MV complex and LV (3).

### Proteome Changes Related to MR

Our cohort presented a large discrepancy of MR severity. Comparison of the MV proteome from patients displaying different levels of MR allowed us to identify candidate proteins and the main biological processes related to MR. First, we compared protein abundance between MVs from patients displaying MSMR and SMR assessed by *vena contracta* measurement, where 20 proteins were found significantly changed. Secondly, we applied a linear correlation relationship between protein abundance in MVs and MR severity, where 26 proteins were found significantly modulated. A total of 39 proteins were found related to MR severity.

Assessment of gene ontology (GO) enrichment associated to these proteins showed biological processes were involved, including glutathione- and reactive oxygen species-related processes. Proteins related to glutathione metabolism (GSTP1 and ESD) are less abundant with the increase of MR severity, suggesting a reduction of glutathione metabolism in the MV as a response to MR. Glutathione metabolism participates in antioxidant defense of the cell. At the same time, we observed an enrichment of the GO “response to reactive oxygen species,” associated with a decrease of PRDX5 in conjunction with MR severity. Altogether, this suggests a reduction of the antioxidant defense system in the MV associated with MR severity.

Assessment of gene ontology enrichment also showed that various inflammatory processes, such as blood coagulation, acute inflammation, IL-1 and IL-12 related processes. Proteins associated with the innate immune response were also found decreased (F12, CAPZA1, A2M, PSME1, HMGB1, CD44), except C3, which was elevated in association with MR severity. Regarding the proteins linked to regulation of coagulation, we detected a decrease of F12, a major member of the coagulation cascade, in combination with an increase of A2M, an inhibitor of thrombin, suggesting a global inhibition of coagulation in the MVs in patients with more severe MR. Enriched inflammatory processes were also associated with decreased protein abundance, suggesting a subside of these pathways, along with MR severity. For instance, proteins linked to the inflammatory response (F12, PRDX, GSTP1), IL-1 production (GSTP1, S100A13, HMGB1), IL-12 response (CFL1 and CAPZA1) were found less abundant in MVs associated with high levels of MR.

Moreover, we observed a decrease of proteins linked to the modulation of peptidase activity (CD44, HMGB1, PSME1, RCN3), as well as an upregulation of an inhibitor of protease (TIMP3) in patients displaying higher MR. Altogether, this suggests a reduction of the proteolytic processes, which could be associated with a late stage of disease. Moreover, our observation linked CD44 to both immune response (GO:0045087) and proteolytic processes (GO:0052547) in the MV. Previous reports showed that CD44 deletion promotes inflammation and reduces collagen degradation during the early phase of cutaneous wound healing, leading to accumulation of collagen even after wound closure (20). We suggest a similar relationship in the MV, leading to the promotion of inflammation and fibrosis.

Overall, our findings indicate that the severity of MR is associated with a decrease of antioxidant defense, proteolysis, coagulation and inflammation modulation.

### Activated Valvular Interstitial Cells

We found a large αSMA+ cell population in IMR MVs. Expression of myofibroblast markers such as αSMA by VICs implies that cells underwent phenotypic modulation from quiescent fibroblasts to activated myofibroblast-like cells (21). The activation of VICs is induced by various stimuli, including abnormal hemodynamic/mechanical forces or soluble factors (22). In our cohort of patients, we observed within the MV samples a strong association between the number of αSMA+ and CD45+ cells, suggesting a relationship between inflammatory cell infiltration and VIC activation. We also noticed a positive correlation of aVIC (αSMA+) numbers with MR severity (*p* = 0.062), albeit insignificant, suggesting an association between these two parameters in IMR. Nevertheless, using a multivariable linear regression model to investigate parameters regulating MR and aVICs showed a significant association with tenting area, but not AL thickness. Mitral tenting area is mainly determined by changes of heart architecture following myocardial infarction (23), suggesting that a myofibroblast-like phenotypic modulation of VICs is regulated by mechanical forces applied on mitral leaflets.

The myofibroblast αSMA+ cell population in the MV could be derived from quiescent VICs, but could also originate from endothelial cells undergoing EndMT by gaining mesenchymal phenotype (24). Further investigations are necessary to determinate the lineage of the aVICs population during IMR.

### Leukocytes

CD45+ cell infiltration within the MV is associated with post-MI remodeling, but in our cohort of patients, we did not observe a correlation of leukocyte infiltration with MR or leaflet thickening. Surgically induced MR in sheep showed that CD45+ cell infiltration was independent of tethering stress, but dependent on occurrence of MI (8). Variation of CD45+ cell infiltration observed in this cohort of post-MI MR could be related to the size of infarcted territory or post-MI time.

CD45 was first shown to be expressed by all hematopoietic lineages, except for erythrocyte and platelets. In MVs, the origin of CD45+ cells co-expressing CD31 or αSMA is not clearly established. One hypothesis suggests bone marrow-derived circulating cells as precursors for different lineages, such as fibrocytes (CD45+ αSMA+) (25) or endothelial progenitor cells (CD45+ CD31+) (26). Nevertheless, the involvement of bone marrow-derived endothelial cells has been associated in humans with vascular endothelial repair or post-ischemia neovascularization (26), but has never been reported in cardiac valve diseases. Secondly, studies from our group showed that primary culture of MV endothelial cells undergoing EndMT after TGFβ treatment was a source of CD45+ cells co-expressing endothelial marker (VE-cadherin) and myofibroblast marker (αSMA) (27). This work demonstrated that CD45 could be a marker of EndMT. Nevertheless, in our study, CD31+ CD45+ and αSMA+ CD45+ cells correspond to a minor portion of mitral cell populations, and do not correlate with MR severity. Biological materials available for this study made it impossible to trace the cell lineage.

### Endothelial-to-Mesenchymal Transition

Previous studies from our group introduced mitral EndMT in a sheep model of MR (8, 9). Here, we demonstrated the presence of CD31+ αSMA+ cells in human MVs with severe IMR, suggesting the occurrence of EndMT. We also examined the association of functional echocardiographic measurement with the rates of EndMT observed in IMR. We detected a close to statistical significance (*p* = 0.052) trend for positive linear correlation of EndMT levels with MR severity. This suggests an association between occurrence of EndMT and MR in humans, as previously described in experimental animal IMR (8, 9, 27). To investigate the parameters regulating MR associated with EndMT, we generated multivariable linear regression models. We observed a significant association between MR severity and combination of EndMT (CD31+ αSMA+) and tenting area or anterior leaflet thickness, indicating a strong relationship affecting MR between the geometric changes induced by mechanical stress applied on the MVs and occurrence of EndMT in humans, as was previously observed in an ovine model (8, 17).

### Prospective New Members Involved in Mitral Valve Endothelial-to-Mesenchymal Transition

A proteomic discovery approach has been used to explore new possible players and biomarkers of EndMT during MR. First, we compared protein abundance between MVs displaying low and high levels of EndMT detected by histo-cytometry. Second, we applied a linear correlation relationship between protein abundance in MVs and EndMT levels. Here, we propose different candidate proteins related to homologous biological processes, such as EMT or EndMT from other vascular beds. The candidate proteins that were not previously associated with EndMT and their hypothetical relationships are presented below.

EndMT is mainly regulated by TGFβ signaling (10). Furthermore, hydrogen peroxide-related oxidative stress potentiates TGFβ-mediated EndMT (28). Analysis of proteomic enrichment associated with EndMT demonstrated an overexpression of proteins implicated in hydrogen peroxide catabolism, including catalase (CAT; high vs. low EndMT, *p* = 0.0045 FC = 2.35; linear correlation to EndMT, *p* = 0.016 *R*^2^ = 0.335), an enzyme responsible for hydrogen peroxide decomposition. Catalase has been described as a critical player in SIRT3-Foxo3a-mediated EndMT in hypertensive renal disease (29). Another enzyme catalyzing the reduction of hydrogen peroxide, Peroxiredoxin-2, was also found increased in correlation with EndMT levels in the MV (PRDX2, linear correlation to EndMT, *p* = 0.038 *R*^2^ = 0.26). Peroxiredoxin-2 was previously described as a potent regulator of TGFβ-mediated EMT (30). We also observed downregulation of an enzyme producing hydrogen peroxide, Monoamine Oxidase A (MAOA, linear correlation to EndMT, *p* = 0.015 *R*^2^ = 0.34), previously shown to be associated with EMT (31). In the context of atherosclerosis, hydrogen peroxide potentiates EndMT and TGFβ-mediated EndMT (28). Conversely, our observations from human MVs showed an increase of hydrogen peroxide catabolism and decrease of its synthesis associated with higher levels of EndMT. This divergence could be attributed to oxidative stress resolution processes or an advanced phase of remodeling.

In addition, we found other proteins related to TGFβ signaling. First, fibrillin 1 (FBN1) was significantly decreased in our samples with high levels of EndMT. Fibrilin 1 is a matrix protein, which forms microfibrils associated with elastic fibers, but also enables them to segregate TGFβ, reducing its bioavailability (32). Fibrilin 1-deficient mice exhibited MV alterations, such as increased length and thickness associated with an increased cell proliferation, decreased apoptosis, and excess TGFβ activation, rescued by TGFβ antagonism (33). This may suggest that the decrease of fibrilin 1 expression is associated with extracellular matrix (ECM) change alongside an increase of TGFβ signaling, increasing EndMT.

Furthermore, we showed a decrease in the abundance of Low-density lipoprotein Receptor Related Protein (LRP1), also called Transforming Growth Factor-β Receptor Type V. LRP1 mediates anti-proliferative effects of TGFβ *in vitro* on various cell types (34, 35). In addition, LRP1 has been shown to promote EMT in prostate cancer cells (36). LRP1-deficient mice presented a Marfan-like syndrome with perturbation of vascular ECM and overactivation of TGFβ signaling (37). While no study focused on cardiac valves in this model, it is reasonable to suggest that decreased levels of LRP1 in human MV could promote cell proliferation and affect the TGFβ pathway.

Some groups already provide hypotheses on extracellular vesicle (EV)-mediated EMT, predominantly in the context of cancer (38, 39), but this regulation has never been established in valve EndMT. Nevertheless, human endothelial cells that underwent EndMT secreted three-times more EVs than control endothelial cells (40). Proteomic analysis of EV content showed enrichment in inflammatory and immune-related proteins (40). These results could partially explain the similar profile of biological process enrichment we observed in leaflets with a high rate of EndMT. This suggests that EndMT, in addition to being induced by inflammatory factors (40), should play a major function in inflammatory progression.

IQGAP1 (IQ motif containing GTPase activating protein 1) was found decreased in MV displaying high level of EndMT and negatively correlated to EndMT level. In endothelial cells, IQGAP1 has been largely described as a key regulator of cell-cell adhesion and cell polarization leading the cell migration (41). EndMT required loss of cell-cell by endothelial cells (42), which is coherent with the downregulation of IQGAP1 related to EndMT observed in MV. Nevertheless, the reduction of IQGAP1 has been associated to an inhibition of EMT in gastric cancer (43), and the clear role of IQGAP1 in EndMT has not been clearly established yet.

Conversely to proteins presented above, which has been observed as actor of EMT or EndMT, some proteins highlighted from proteomics have never been described in mesenchymal-like phenotype transitioning process. These new candidate proteins may reveal hypothetical mechanism implicated in EndMT regulation in MR.

Erythrocyte's proteins HBA2, HBB, HBD (respectively hemoglobin alpha2, beta, delta) and GYPA (glycophorin A), were found overexpress in MVs displaying high level of EndMT, as well as linearly correlated to EndMT level. In the context of atherosclerosis, studies have demonstrated an infiltration of erythrocytes within the atheromatous lesion (44), promoting oxidative processes by the release of Redox-active ferrous iron (Fe++) (45, 46). In calcified aortic valve, Morvan et al. observed erythrocytes and iron deposits into the fibrosa related to endothelial fissure *in vivo*, leading to change of VICs differentiation into a pro-inflammatory and osteogenic phenotype (47). Occurrence of endothelial fissure and erythrocyte infiltration have not been shown in MR. Nevertheless, endothelial dysfunctions post-MR have been described, such as reduction of antioxidant ability, increase of osteoprogerin secretion and higher level of circulating microparticle (48). Altogether, these data suggest a plausible endothelial dysfunction associated to an erythrocyte infiltration, leading to an increase of oxidative stress within mitral leaflets. Furthermore, FTL (ferritin light chain) is decreased in mitral leaflets presenting high level of EndMT. This protein is an iron storage protein and critical for the protection again iron-mediated oxidative damage as well as inflammation (49). The decrease of FTL observed here imply a positive relationship between iron toxicity and level of EndMT. Nevertheless, no direct evidence has been established so far.

PPBP (pro-platelet basic protein), also called CXCL7, has been found positively correlated to EndMT level in mitral leaflets (*R* = 0.64, *p* = 0.0057). It is a chemokine released by alpha granule platelets, which signals through binding to its receptor CXCR2. Other chemokine, such as CXCL5 has been described to promote EMT of nasopharyngeal carcinoma cells by activating ERK/GSK-3β/snail signaling (50), and previous report mentioned that PPBP is able to promote proliferation and invasion of carcinoma cells (51), suggesting the possibility of an PPBP-regulated mesenchymal transition. Additionally, Grande et al. observed in platelet-derived microparticles from obese patients an enhanced capacity to induce EMT and EndMT marker genes *in vitro*. In addition, they found in these platelet-derived microparticles an overexpression of a few proteins, including PPBP (52). However, direct implication of PPBP in EndMT has not yet be determined.

TTN (titin) was found overexpressed in MV presenting high levels of EndMT. TTN is important for sarcomere contraction of muscle cells but were also described in smooth muscle cell and non-muscle cells in stress fibers (53). In addition, a nuclear form of titin has described in non-muscle cell as a key factor in laminin adhesion for nuclear organization (54). Subsequently, possible implication of TTN in EndMT might involve cytoskeleton reorganization through the stress fiber or epigenetic regulation through its nuclear form.

CD55, also known as complement decay-accelerating factor, was found decreased in mitral leaflet displaying high level of EndMT. Primary function of CD55 is to prevent the complement system through inhibition of C3 and C5 convertase, responsible of proteolytic cleavage of C3 into C3a and C3b and C5 into C5a and C5b. Interestingly, it has been shown that activation of C3aR is important for EMT in renal epithelium as well as in ovarian cancer cells (55). Furthermore, endothelial cells express C3aR and C5aR, leading to proliferation, actin cytoskeleton response or chemotaxis (55, 56).

Assessment of proteomic regulation corroborated with histo-cytomety measurement in mitral leaflets allowed to established gene ontology (GO) enrichment associated to EndMT, including oxidative stress, inflammation as well as vesicle-related processes. Furthermore, identification of new candidate proteins associated with EndMT permitted to present plausible new biological processes involved in MV's EndMT, such as iron-mediated oxidative process, platelets-derived vesicles or complement system. Nevertheless, further investigations are required to validate candidate proteins and establish their direct implication in EndMT during MR.

## Limitations

For our study, patients' clinical history is unknown and could represent confounding factors such as medication history, co-morbidities, or proximity to initial MI. For instance, ACEi/ARB medications are likely to affect the histological characteristics of the valves and the severity of mitral regurgitation (9, 57). In addition, location and size of the primary cardiac infarction is unknown, which could also be a confounding factor regulating heart architecture post-MI. Furthermore, the small size of our cohort limited the statistical analysis and generation of univariable and multivariable regression model. However, gender representation is close to parity (Male: 58.8%; Female: 41.2%). Finally, the use of snap frozen human samples limited our cell phenotyping strategy for the identification of cell populations through immunofluorescence, including cells undergoing EndMT. Nevertheless, this study has provided us with a rare opportunity to analyze mitral valves from a unique population of patient with IMR.

## Conclusion

Little is known about the mechanism of MV adaptation to ischemic heart remodeling in humans. Our investigation on a small cohort of patients with IMR provides an overall comprehension of general biological processes implicated as well as new cell population correlated with the severity of the regurgitation. For the first time in human MVs, we highlight the presence of EndMT during IMR, and its close relationship with MR severity, leaflet thickening and tenting. Moreover, our study corroborated observations from animal models of IMR and opens now the perspective of validation of candidate proteins for key functional actor or biomarker with *in vitro* approach and an independent cohort.

## Data Availability Statement

The datasets presented in this study can be found in online repositories. The names of the repository/repositories and accession number(s) can be found at: https://www.ebi.ac.uk/pride/archive/, PXD025096.

## Author Contributions

AL, YN, KK, EA, JB, and RL contributed to conception and design of the study. AL, YN, and KK performed the statistical analysis. HH and SS performed mass spectrometry and organized the proteomic database. AL wrote the manuscript. YN wrote sections of the manuscript. EA, JB, and RL edited and critically revised manuscript and contributed to overall project supervision and funding. All authors contributed to manuscript revision, read, and approved the submitted version.

## Author Disclaimer

The views expressed in this article are those of the authors and do not necessarily represent the views of the National Heart, Lung and Blood Institute, the National Institute of Neurological Disorders and Stroke, the National Institutes of Health, or the US Department of Health and Human Services.

## Conflict of Interest

The authors declare that the research was conducted in the absence of any commercial or financial relationships that could be construed as a potential conflict of interest.

## Publisher's Note

All claims expressed in this article are solely those of the authors and do not necessarily represent those of their affiliated organizations, or those of the publisher, the editors and the reviewers. Any product that may be evaluated in this article, or claim that may be made by its manufacturer, is not guaranteed or endorsed by the publisher.
