# Supplementary material for: Integration of Functional Imaging, Cytometry, and Unbiased Proteomics Reveals New Features of Endothelial-to-Mesenchymal Transition in Ischemic Mitral Valve Regurgitation in Human Patients
Source: Front Cardiovasc Med. 2021 Aug 12;8:688396. doi: 10.3389/fcvm.2021.688396 (PMC8387660; doi:10.3389/fcvm.2021.688396)
Supplement: Supplementary file 1 [file Data_Sheet_1.PDF]

Supplementary Figure 1

A

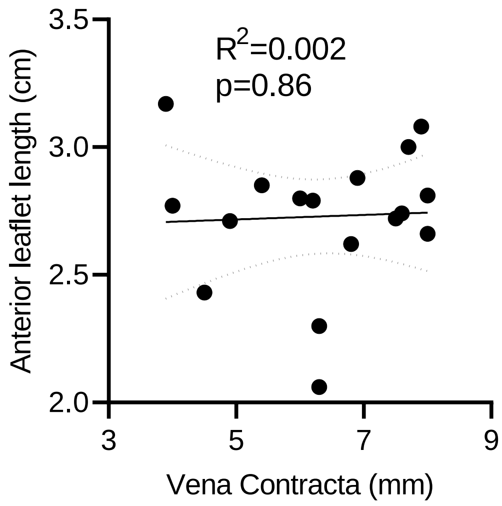

Supplementary Figure 1. Linear correlation between *vena contracta* and anterior leaflet length.

Supplementary Figure 2

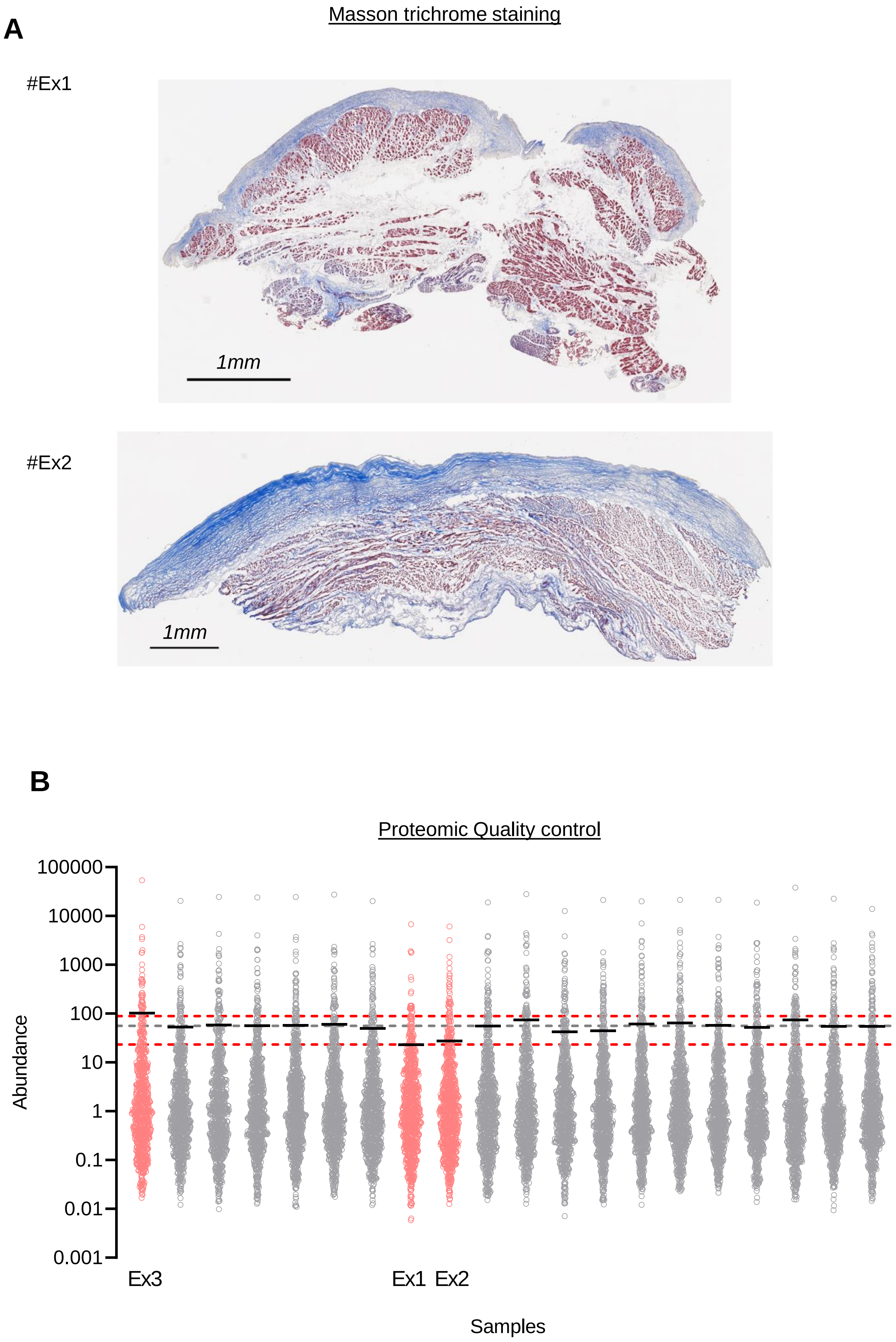

Supplementary Figure 2. A Masson trichrome stained cryosection of excluded biopsy (Ex1 and Ex2) showing in red the high density of muscular fiber, implying the non-valvular structure of this tissue. **B** Normalized abundance of proteins identified from each sample. Black bars indicate mean of protein the abundance for each sample. Red dotted lines represent the confidence interval of 2 standard deviations of the abundance means. Pink violin plots indicate excluded sample (Ex1, Ex2, Ex3).

Supplementary Figure 3

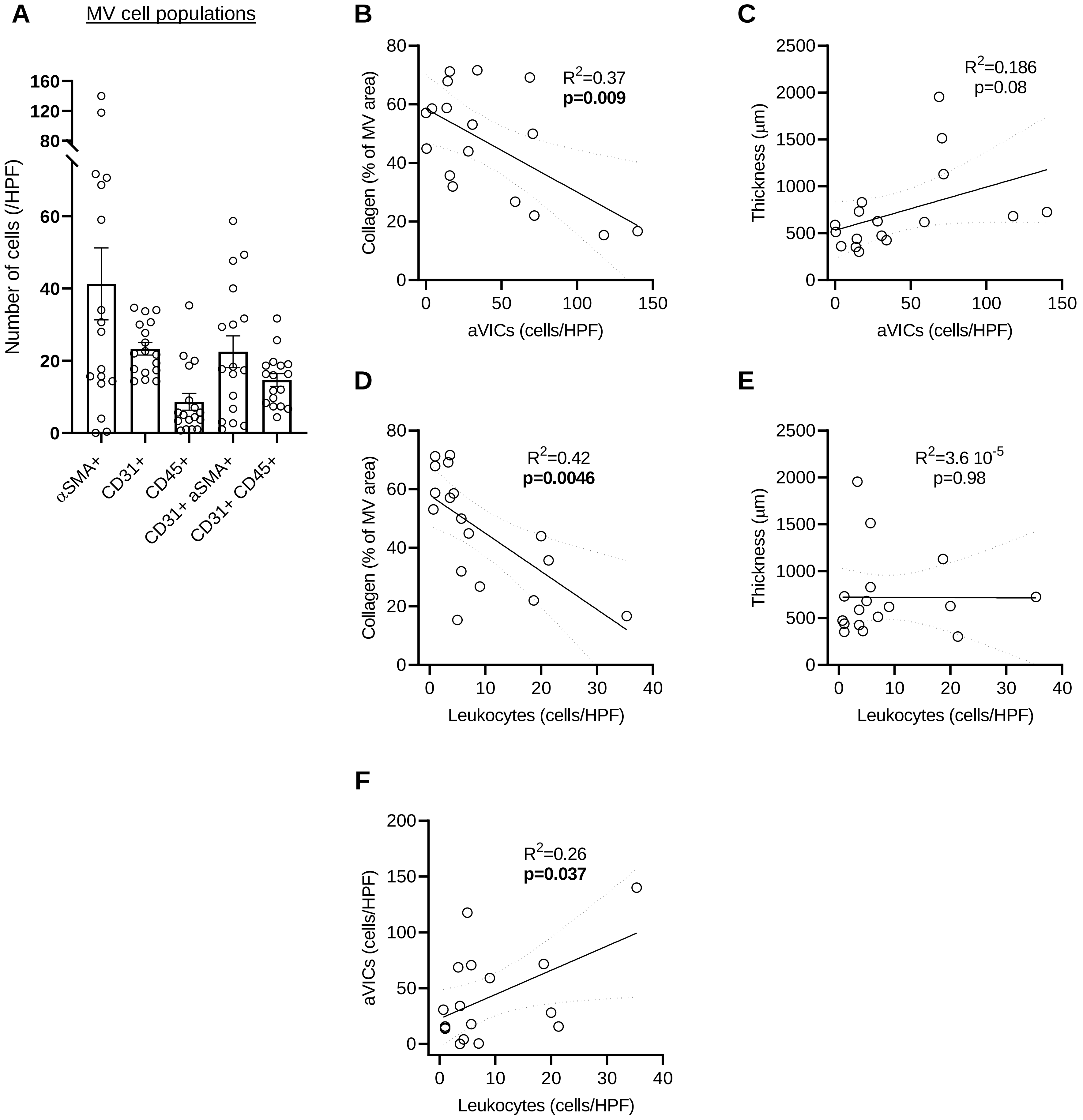

Supplementary Figure 3. Linear regression between αSMA+ activated valvular interstitial cells (aVICs) and MV thickness (**A**) or Collagen content of MV (**B**). Linear regression between CD45+ leukocyte and MV thickness (**C**) or Collagen content of MV (**D**). **E** Correlation between Leukocytes and aVICs content of MV. Thin dotted line shows the 95% confidence interval.

Supplementary Figure 4

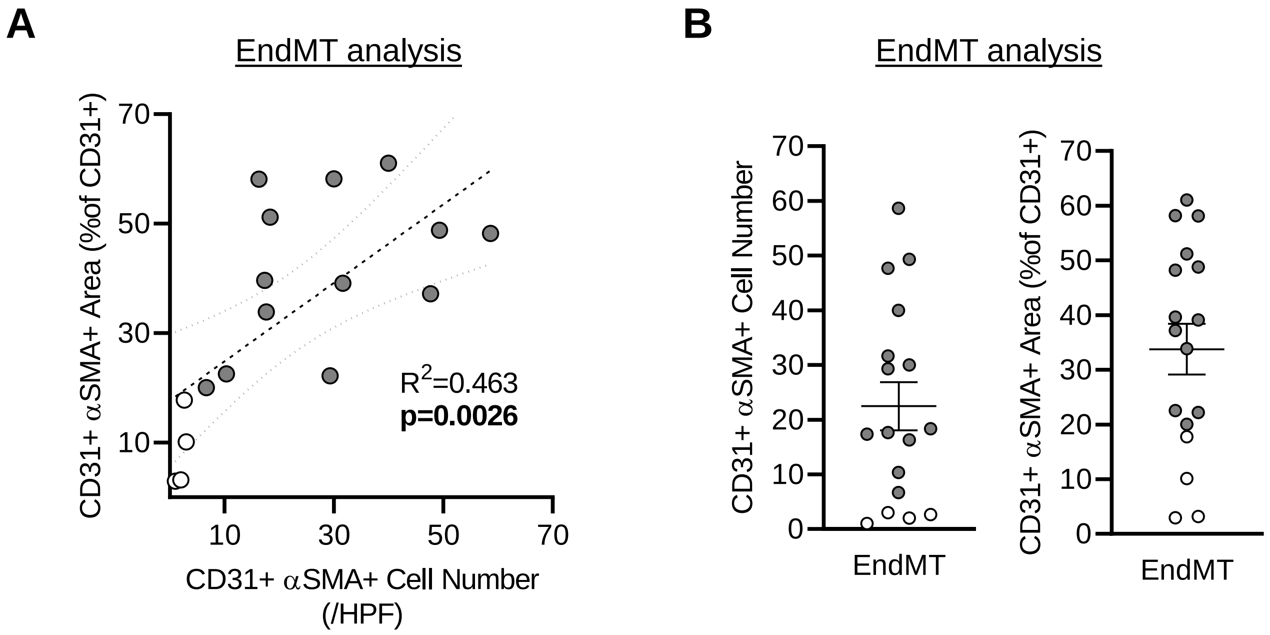

Supplementary Figure 4. A Level of EndMT is estimated using 2 methods: Counting of CD31+ αSMA+ cells and CD31+ αSMA+ double positive area normalized to total CD31+ area. Dot plot display the correspondence between both quantification methods shows a significant linear correlation (R^2=0.463, p=0.0026), thin dotted line indicates the 95% confidence interval. Samples having low level of EndMT are represented by white dots, and samples with higher level of EndMT are represented by grey dots.
