# Supplementary material for: Integration of Functional Imaging, Cytometry, and Unbiased Proteomics Reveals New Features of Endothelial-to-Mesenchymal Transition in Ischemic Mitral Valve Regurgitation in Human Patients
Source: Front Cardiovasc Med. 2021 Aug 12;8:688396. doi: 10.3389/fcvm.2021.688396 (PMC8387660; doi:10.3389/fcvm.2021.688396)
Supplement: Supplementary file 2 [file Data_Sheet_2.docx]

Supplementary Tables

# Supplementary Table 1

| **Sample ID** | **Vena contracta (mm)** | **AL thickness (mm)** | **PL thickness (mm)** | **AL length**  **(cm)** | **PL length**  **(cm)** | **Tenting area (cm^2^)** |
| --- | --- | --- | --- | --- | --- | --- |
| 1 | 5.4 | 2.1 | 2 | 2.85 | 1.82 | 2.63 |
| 2 | 6.3 | 1.8 | 2.6 | 2.3 | 1.81 | 1.97 |
| 3 | 4.9 | 1.7 | 1.4 | 2.71 | 1.82 | 2.17 |
| 4 | 6 | 2.2 | 2 | 2.8 | 2.17 | 3.38 |
| 5 | 4.5 | 1.5 | 2 | 2.43 | 1.42 | 1.05 |
| 6 | 6.2 | 1.9 | 2.1 | 2.79 | 1.75 | 2.18 |
| 7 | 4 | 1.9 | 2.3 | 2.77 | 1.78 | 1.63 |
| 8 | 6.9 | 1.5 | 1.6 | 2.88 | 1.69 | 2.54 |
| 9 | 6.3 | 2.3 | 2 | 2.06 | 1.38 | 1.29 |
| 10 | 8 | 2.1 | 2 | 2.81 | 2.81 | 5.07 |
| 11 | 8 | 2.7 | 2.7 | 2.66 | 1.92 | 2.33 |
| 12 | 7.9 | 2.4 | 2 | 3.08 | 1.4 | 2.68 |
| 13 | 7.7 | 2.6 | 2.5 | 3 | 1.77 | 1.93 |
| 14 | 3.9 | 1.4 | 1.4 | 3.17 | 1.66 | 1.55 |
| 15 | 6.8 | 2.1 | 2.2 | 2.62 | 1.88 | 1.95 |
| 16 | 7.5 | 1.6 | 1.7 | 2.72 | 1.64 | 2.21 |
| 17 | 7.6 | 1.7 | 2 | 2.74 | 2.12 | 2.59 |
| **Average** | 6.347059 | 1.970588 | 2.029412 | 2.728824 | 1.814118 | 2.302941 |
| **SD** | 1.395581 | 0.38853 | 0.370413 | 0.272004 | 0.337232 | 0.909559 |

**Supplementary Table 1.** Table of echocardiographic measures for each donor, assessed before surgery.

- **Supplementary Table 2**

| **Sample ID** | **Age (years)** | **Gender** | **Thickness (µm)** |
| --- | --- | --- | --- |
| 1 | 55 | M | 587.6 |
| 2 | 75 | F | 682.7 |
| 3 | 56 | F | 725.9 |
| 4 | 75 | F | 619 |
| 5 | 57 | M | 628.7 |
| 6 | 64 | F | 439.9 |
| 7 | 73 | F | 353.3 |
| 8 | 60 | M | 425.3 |
| 9 | 53 | F | 362.5 |
| 10 | 75 | M | 474.2 |
| 11 | 67 | M | 1513.9 |
| 12 | 51 | M | 731.5 |
| 13 | 65 | M | 1129.2 |
| 14 | 72 | M | 302.4 |
| 15 | 85 | M | 1954.8 |
| 16 | 69 | M | 514.2 |
| 17 | 53 | F | 830.3 |
| **Median** | 65 | N/A | 619 |
| **Average** | 65 | 58.8% M | 722.0824 |
| **SD** | 9.962429 | N/A | 438.5281 |

**Supplementary Table 2.** Table of age (years), gender (male, M or female, F) and mitral valve thickness (µm) for each donor from biopsy sample. N/A, not applicable.

- **Supplementary Table 3**

| **MSMR vs SMR** | | | |  | **Linear Correlation to MR** | | | | |
| --- | --- | --- | --- | --- | --- | --- | --- | --- | --- |
| **Gene_name** | **p value** | **Fold Change** | **Log2(FC)** |  | **Gene_name** | **R** | **R^2** | **P value** |  |
| **MDGA2** | **0.017478** | **2.010091** | **1.007261** |  | ECH1 | 0.59601 | 0.355228 | 0.007475 |  |
| **PLEKHC1** | **0.039405** | **1.836055** | **0.876609** |  | A2M | 0.56665 | 0.321092 | 0.011933 |  |
| CHST14 | 0.039552 | 1.483669 | 0.569169 |  | **MDGA2** | **0.522018** | **0.272503** | **0.022582** |  |
| EIF4A2 | 0.042006 | 1.531822 | 0.615249 |  | ACADS | 0.517255 | 0.267553 | 0.02406 |  |
| CHAD | 0.042706 | 2.063737 | 1.045259 |  | CASQ2 | 0.504815 | 0.254838 | 0.028282 |  |
| TIMP3 | 0.04413 | 1.627907 | 0.703018 |  | **PLEKHC1** | **0.501716** | **0.251719** | **0.029419** |  |
|  |  |  |  |  | ACO2 | 0.48079 | 0.231159 | 0.038062 |  |
| FUS | 0.006459 | 0.593994 | -0.75148 |  | C3 | 0.478469 | 0.228932 | 0.03913 |  |
| HMGB1 | 0.015343 | 0.469609 | -1.09047 |  |  |  |  |  |  |
| **F12** | **0.015754** | **0.467328** | **-1.09749** |  | **GC** | **-0.68878** | **0.474414** | **0.001228** |  |
| **PRDX5** | **0.018924** | **0.748545** | **-0.41784** |  | **ESD** | **-0.65075** | **0.423473** | **0.00276** |  |
| HNRPD | 0.019811 | 0.705124 | -0.50405 |  | **F12** | **-0.62227** | **0.387226** | **0.004738** |  |
| SRSF2 | 0.024799 | 0.348765 | -1.51967 |  | **PRDX5** | **-0.61572** | **0.379106** | **0.005328** |  |
| PSME1 | 0.025119 | 0.700362 | -0.51383 |  | PLIN1 | -0.59702 | 0.356434 | 0.00735 |  |
| **GC** | **0.026087** | **0.312067** | **-1.68007** |  | **GSTP1** | **-0.59591** | **0.35511** | **0.007487** |  |
| **ESD** | **0.028415** | **0.759679** | **-0.39654** |  | GATM | -0.56896 | 0.323711 | 0.011519 |  |
| SFRS3 | 0.02979 | 0.238953 | -2.0652 |  | SH3BGRL3 | -0.5669 | 0.321377 | 0.011887 |  |
| PDIA3 | 0.041616 | 0.798236 | -0.32511 |  | HNRNPH3 | -0.53786 | 0.289291 | 0.018176 |  |
| **GSTP1** | **0.041984** | **0.779564** | **-0.35926** |  | CFL1 | -0.5229 | 0.273421 | 0.022317 |  |
| SEPTIN2 | 0.043007 | 0.607268 | -0.71959 |  | TNC | -0.51957 | 0.269956 | 0.023332 |  |
|  |  |  |  |  | RCN3 | -0.49987 | 0.249866 | 0.030115 |  |
|  |  |  |  |  | GFAP | -0.4881 | 0.238241 | 0.034845 |  |
|  |  |  |  |  | PPP1R7 | -0.47928 | 0.229709 | 0.038754 |  |
|  |  |  |  |  | CAPZA1 | -0.47803 | 0.228512 | 0.039335 |  |
|  |  |  |  |  | S100A13 | -0.46517 | 0.21638 | 0.045706 |  |
|  |  |  |  |  | CD44 | -0.46364 | 0.214964 | 0.04651 |  |
|  |  |  |  |  | PCOLCE | -0.458 | 0.209763 | 0.049583 |  |

**Supplementary Table 3.** Tables of proteins with significantly modulated in SMR samples compared to MSMR samples (left) and correlated with MR severity (right). Proteins with increased expression (Log2FC>0 or R>0) are shown in red, while the proteins with significantly decreased expression (Log2FC<0 or R<0) are presented in blue. Proteins shared in both analyses are presented in bold.

- **Supplementary Table 4**

| **High vs Low EndMT** | | | |  | **Linear Correlation to EndMT** | | | |
| --- | --- | --- | --- | --- | --- | --- | --- | --- |
| **Gene_name** | **p value** | **Fold Change** | **Log2(FC)** |  | **Gene_name** | **R** | **R^2** | **P value** |
| MED17 | 0.000899023 | 2.147185218 | 1.102446644 |  | PPBP | 0.643936173 | 0.414653795 | 0.005701 |
| JCHAIN | 0.001593409 | 2.593417993 | 1.374854751 |  | **CA2** | **0.613433222** | **0.376300317** | **0.009395** |
| **HBA2** | **0.003429675** | **6.193375251** | **2.63072586** |  | **CAT** | **0.579120077** | **0.335380064** | **0.015607** |
| **CA2** | **0.004421777** | **3.315962123** | **1.729427527** |  | **EHD2** | **0.569780489** | **0.324649805** | **0.017761** |
| **CAT** | **0.004499654** | **2.347036833** | **1.230840483** |  | **HRG** | **0.563338715** | **0.317350507** | **0.019378** |
| MAP4 | 0.004613701 | 1.995183972 | 0.996521781 |  | **HBD** | **0.541436867** | **0.293153881** | **0.025754** |
| SNCA | 0.005047303 | N/A | N/A |  | **GYPA** | **0.53432483** | **0.285503024** | **0.02814** |
| APOA1 | 0.005365771 | 1.585162245 | 0.664630511 |  | HAGH | 0.521068646 | 0.271512534 | 0.03304 |
| **HBD** | **0.005820663** | **4.105658318** | **2.037613568** |  | **CAVIN3** | **0.50856827** | **0.258641685** | **0.038238** |
| **HRG** | **0.005956874** | **1.670466376** | **0.740250944** |  | PRDX2 | 0.508252775 | 0.258320883 | 0.038377 |
| COL1A1 | 0.006050014 | 2.093729975 | 1.066075392 |  | **HBB** | **0.496053877** | **0.246069449** | **0.044044** |
| FBLIM1 | 0.008248391 | 3.507511389 | 1.810447789 |  | **HBA2** | **0.491808905** | **0.241875999** | **0.046158** |
| TTN | 0.008713797 | 1.511941978 | 0.596402776 |  | BLVRB | 0.486543367 | 0.236724448 | 0.048886 |
| **HBB** | **0.009264079** | **3.627484134** | **1.858969304** |  | LAMA5 | 0.480855089 | 0.231221617 | 0.038032 |
| HMGB1 | 0.010927109 | 2.145507286 | 1.1013188 |  | PRDX6 | 0.459635677 | 0.211264956 | 0.048676 |
| SPTB | 0.011142539 | 10.83367948 | 3.437451409 |  |  |  |  |  |
| SLC4A1 | 0.011811073 | 9.356986027 | 3.226043899 |  | IGH | -0.459846187 | 0.211458516 | 0.048561 |
| CA1 | 0.013606055 | 3.445348498 | 1.78464992 |  | SFPQ | -0.464387477 | 0.215655729 | 0.046115 |
| CAST | 0.013780092 | 1.596524352 | 0.674934559 |  | LAMB1 | -0.464936231 | 0.216165699 | 0.045826 |
| CLIC5 | 0.015534076 | N/A | N/A |  | CYBRD1 | -0.466625258 | 0.217739132 | 0.044946 |
| RPS12 | 0.01599671 | 2.029199068 | 1.020910403 |  | LAMP2 | -0.467674938 | 0.218719848 | 0.044405 |
| TAGLN2 | 0.018460054 | 1.554310929 | 0.636275133 |  | FBLN2 | -0.468926021 | 0.219891613 | 0.043767 |
| **GYPA** | **0.01854122** | **7.724053907** | **2.949358233** |  | HSPA2 | -0.4716133 | 0.222419105 | 0.042421 |
| NID1 | 0.020393587 | 2.068630247 | 1.048675797 |  | ESD | -0.472664268 | 0.22341151 | 0.041903 |
| LIMS2 | 0.023282378 | 7.907813399 | 2.983278828 |  | CAPZA1 | -0.473675084 | 0.224368085 | 0.041409 |
| EPB41L2 | 0.023363845 | 2.535781753 | 1.342430583 |  | UQCRC1 | -0.475041794 | 0.225664706 | 0.040749 |
| FGL2 | 0.027229366 | 1.657437485 | 0.728954456 |  | KCTD12 | -0.475993156 | 0.226569484 | 0.040295 |
| PDLIM5 | 0.028214485 | 1.630707801 | 0.705498295 |  | HSP90AB1 | -0.483769592 | 0.234033018 | 0.036724 |
| **CAVIN3** | **0.029479097** | **1.739270124** | **0.798482014** |  | PLXDC2 | -0.486093087 | 0.236286489 | 0.049125 |
| SRSF2 | 0.03108506 | 2.956425374 | 1.563853861 |  | **NDUFA7** | **-0.495469174** | **0.245489702** | **0.044331** |
| HDGF | 0.031411084 | 1.537978896 | 0.621035707 |  | LDHA | -0.496631586 | 0.246642932 | 0.043762 |
| TGFB1I1 | 0.032061869 | 3.623747846 | 1.857482571 |  | CPB2 | -0.507943453 | 0.258006552 | 0.038513 |
| AFM | 0.032948589 | 1.498881782 | 0.583886601 |  | SRPX | -0.508689154 | 0.258764656 | 0.038185 |
| HSPB2 | 0.03373806 | N/A | N/A |  | **LRP1** | **-0.516633774** | **0.266910457** | **0.034818** |
| SRSF1 | 0.035560422 | 11.04742142 | 3.465637764 |  | **HNRNPH3** | **-0.518557942** | **0.268902339** | **0.034038** |
| LASP1 | 0.035938837 | 3.799906511 | 1.925963925 |  | PGLS | -0.522298287 | 0.2727955 | 0.03256 |
| **EHD2** | **0.036024974** | **1.463282256** | **0.549208082** |  | PLA2G2A | -0.527974823 | 0.278757414 | 0.030412 |
| LMCD1 | 0.036780746 | 2.254779742 | 1.172986511 |  | A0A140VJL8 | -0.52860182 | 0.279419884 | 0.030181 |
| APOC4-APOC2 | 0.03739955 | 2.573117175 | 1.363517156 |  | **IQGAP1** | **-0.531526363** | **0.282520274** | **0.029124** |
| H1FX | 0.038091633 | 1.841148121 | 0.880605697 |  | ERP70 | -0.533252275 | 0.284357989 | 0.028514 |
| IGKV2D-24 | 0.039883319 | 1.971528082 | 0.97931426 |  | BST1 | -0.537352754 | 0.288747982 | 0.027104 |
| KRT14 | 0.04060256 | 4.13766912 | 2.048818281 |  | CNDP2 | -0.540778147 | 0.292441004 | 0.025968 |
| TINAGL1 | 0.045095955 | 1.986427701 | 0.990176285 |  | RBMX | -0.542015215 | 0.293780494 | 0.025567 |
| HINT1 | 0.046340935 | 1.749799008 | 0.807189216 |  | SERPINB6 | -0.542754387 | 0.294582325 | 0.025329 |
| MYL6 | 0.047474147 | 2.391365491 | 1.257834645 |  | ADD3 | -0.547167769 | 0.299392567 | 0.023948 |
| SFRS3 | 0.049673069 | 3.151652151 | 1.656108313 |  | F13A1 | -0.553152355 | 0.305977528 | 0.022168 |
|  |  |  |  |  | FTH1 | -0.555322179 | 0.308382722 | 0.021549 |
| APCS | 0.005364839 | 0.636176922 | -0.652500057 |  | CLIC1 | -0.577774225 | 0.333823055 | 0.015904 |
| TUFM | 0.009890313 | 0.660045672 | -0.599362239 |  | MAOA | -0.582424588 | 0.3392184 | 0.014896 |
| **NDUFA7** | **0.01103348** | **0.288608593** | **-1.79281384** |  | SCARB2 | -0.588760003 | 0.346638341 | 0.013605 |
| ARF4 | 0.016077432 | 0.692362607 | -0.530400285 |  | ECI2 | -0.614641077 | 0.377783653 | 0.00922 |
| HSPD1 | 0.017071341 | 0.795066399 | -0.330852745 |  | **LAP3** | **-0.633310859** | **0.401082645** | **0.006823** |
| **FTH1** | **0.018443415** | **0.437106546** | **-1.19394311** |  |  |  |  |  |
| **LRP1** | **0.020007404** | **0.702503063** | **-0.50942358** |  |  |  |  |  |
| FTL | 0.021039818 | 0.561581474 | -0.83243275 |  |  |  |  |  |
| ANXA4 | 0.029725421 | 0.656644091 | -0.606816471 |  |  |  |  |  |
| **IQGAP1** | **0.038593985** | **0.557545717** | **-0.842837988** |  |  |  |  |  |
| **HNRNPH3** | **0.040379214** | **0.745068645** | **-0.424554743** |  |  |  |  |  |
| **LAP3** | **0.041456732** | **0.393720929** | **-1.344754691** |  |  |  |  |  |
| FBN1 | 0.042672237 | 0.814095917 | -0.296729311 |  |  |  |  |  |
| CD55 | 0.049931265 | 0.407331119 | -1.295726058 |  |  |  |  |  |

**Supplementary Table 4.** Tables of proteins with significantly modulated in samples displaying high level of EndMT compared to samples low level of EndMT (left) and correlated with EndMT rate (right). Proteins with increased expression (Log2FC>0 or R>0) are shown in red, while the proteins with significantly decreased expression (Log2FC<0 or R<0) are presented in blue. Proteins shared in both analyses are presented in bold. N/A, not applicable.

- **Supplementary Table 5**

| **Gene_name** | **Endothelial-to-mesenchymal transition** | **Epithelial-to-mesenchymal transition** |
| --- | --- | --- |
| HRG |  | [1] |
| EHD2 |  | [2] |
| FGL2 |  | [3] |
| FBN1 |  | [4; 5] |
| CA2 |  | [6] |
| LASP1 |  | [7; 8] |
| HMGB1 |  | [9] |
| LMCD1 |  | [10; 11] |
| NID1 |  | [12] |
| FTH1 |  | GO0001837, [13] |
| TGFB1I1 |  | GO0001837 |
| PRDX2 |  | [14] |
| SRPX |  | [15] |
| LRP1 |  | [16] |
| PLA2G2A |  | [17] |
| MAOA |  | [18] |
| TTN |  | [19] |
| IQGAP1 |  | [20; 21] |
| COL1A1 | [22] |  |
| CAST | [23] |  |
| TAGLN2 | [24] |  |
| CAT | [25] |  |
| APOA1 | [26] |  |
| BLVRB | [27] |  |

[1] J. Cedervall, Y. Zhang, M. Ringvall, A. Thulin, A. Moustakas, W. Jahnen-Dechent, A. Siegbahn, and A.K. Olsson, HRG regulates tumor progression, epithelial to mesenchymal transition and metastasis via platelet-induced signaling in the pre-tumorigenic microenvironment. Angiogenesis 16 (2013) 889-902.

[2] Y. Shi, X. Liu, Y. Sun, D. Wu, A. Qiu, H. Cheng, C. Wu, and X. Wang, Decreased expression and prognostic role of EHD2 in human breast carcinoma: correlation with E-cadherin. J Mol Histol 46 (2015) 221-31.

[3] W.Z. Qin, Q.L. Li, W.F. Chen, M.D. Xu, Y.Q. Zhang, Y.S. Zhong, L.L. Ma, J.W. Hu, M.Y. Cai, M.J. He, L.Q. Yao, and P.H. Zhou, Overexpression of fibrinogen-like protein 2 induces epithelial-to-mesenchymal transition and promotes tumor progression in colorectal carcinoma. Med Oncol 31 (2014) 181.

[4] H.C. Lien, Y.H. Lee, Y.L. Juang, and Y.T. Lu, Fibrillin-1, a novel TGF-beta-induced factor, is preferentially expressed in metaplastic carcinoma with spindle sarcomatous metaplasia. Pathology 51 (2019) 375-383.

[5] A.K. Kiemer, K. Takeuchi, and M.P. Quinlan, Identification of genes involved in epithelial-mesenchymal transition and tumor progression. Oncogene 20 (2001) 6679-88.

[6] C. Zhang, H. Wang, Z. Chen, L. Zhuang, L. Xu, Z. Ning, Z. Zhu, P. Wang, and Z. Meng, Carbonic anhydrase 2 inhibits epithelial-mesenchymal transition and metastasis in hepatocellular carcinoma. Carcinogenesis 39 (2018) 562-570.

[7] H. Wang, J. Shi, Y. Luo, Q. Liao, Y. Niu, F. Zhang, Z. Shao, Y. Ding, and L. Zhao, LIM and SH3 protein 1 induces TGFbeta-mediated epithelial-mesenchymal transition in human colorectal cancer by regulating S100A4 expression. Clin Cancer Res 20 (2014) 5835-47.

[8] E. Butt, and D. Raman, New Frontiers for the Cytoskeletal Protein LASP1. Front Oncol 8 (2018) 391.

[9] J. Zhang, S. Shao, D. Han, Y. Xu, D. Jiao, J. Wu, F. Yang, Y. Ge, S. Shi, Y. Li, W. Wen, and W. Qin, High mobility group box 1 promotes the epithelial-to-mesenchymal transition in prostate cancer PC3 cells via the RAGE/NF-kappaB signaling pathway. Int J Oncol 53 (2018) 659-671.

[10] L. Du, S. Yamamoto, B.L. Burnette, D. Huang, K. Gao, N. Jamshidi, and M.D. Kuo, Transcriptome profiling reveals novel gene expression signatures and regulating transcription factors of TGFbeta-induced epithelial-to-mesenchymal transition. Cancer Med 5 (2016) 1962-72.

[11] U. Valcourt, M. Kowanetz, H. Niimi, C.H. Heldin, and A. Moustakas, TGF-beta and the Smad signaling pathway support transcriptomic reprogramming during epithelial-mesenchymal cell transition. Mol Biol Cell 16 (2005) 1987-2002.

[12] Y. Zhou, Y. Zhu, X. Fan, C. Zhang, Y. Wang, L. Zhang, H. Zhang, T. Wen, K. Zhang, X. Huo, X. Jiang, Y. Bu, and Y. Zhang, NID1, a new regulator of EMT required for metastasis and chemoresistance of ovarian cancer cells. Oncotarget 8 (2017) 33110-33121.

[13] N. Lobello, F. Biamonte, M.E. Pisanu, M.C. Faniello, Z. Jakopin, E. Chiarella, E.D. Giovannone, R. Mancini, G. Ciliberto, G. Cuda, and F. Costanzo, Ferritin heavy chain is a negative regulator of ovarian cancer stem cell expansion and epithelial to mesenchymal transition. Oncotarget 7 (2016) 62019-62033.

[14] J. Feng, Z. Fu, J. Guo, W. Lu, K. Wen, W. Chen, H. Wang, J. Wei, and S. Zhang, Overexpression of peroxiredoxin 2 inhibits TGF-beta1-induced epithelial-mesenchymal transition and cell migration in colorectal cancer. Mol Med Rep 10 (2014) 867-73.

[15] M.Y. Feng, K. Wang, Q.T. Shi, X.W. Yu, and J.S. Geng, Gene expression profiling in TWIST-depleted gastric cancer cells. Anat Rec (Hoboken) 292 (2009) 262-70.

[16] M.W. Hance, K. Dole, U. Gopal, J.E. Bohonowych, A. Jezierska-Drutel, C.A. Neumann, H. Liu, I.P. Garraway, and J.S. Isaacs, Secreted Hsp90 is a novel regulator of the epithelial to mesenchymal transition (EMT) in prostate cancer. J Biol Chem 287 (2012) 37732-44.

[17] C. Wu, J. Su, X. Wang, J. Wang, K. Xiao, Y. Li, Q. Xiao, M. Ling, Y. Xiao, C. Qin, W. Long, F. Zhang, Y. Pan, F. Xiang, and Q. Liu, Overexpression of the phospholipase A2 group V gene in glioma tumors is associated with poor patient prognosis. Cancer Manag Res 11 (2019) 3139-3152.

[18] F. Liu, L. Hu, Y. Ma, B. Huang, Z. Xiu, P. Zhang, K. Zhou, and X. Tang, Increased expression of monoamine oxidase A is associated with epithelial to mesenchymal transition and clinicopathological features in non-small cell lung cancer. Oncol Lett 15 (2018) 3245-3251.

[19] Y. Jia, Y. Duan, T. Liu, X. Wang, W. Lv, M. Wang, J. Wang, and L. Liu, LncRNA TTN-AS1 promotes migration, invasion, and epithelial mesenchymal transition of lung adenocarcinoma via sponging miR-142-5p to regulate CDK5. Cell Death Dis 10 (2019) 573.

[20] J. Liu, X. Ni, Y. Li, M. Chen, W. Chen, Y. Wu, B. Chen, Y. Wu, and M. Xu, Downregulation of IQGAP1 inhibits epithelial-mesenchymal transition via the HIF1alpha/VEGF-A signaling pathway in gastric cancer. J Cell Biochem 120 (2019) 15790-15799.

[21] W. Hu, Z. Wang, S. Zhang, X. Lu, J. Wu, K. Yu, A. Ji, W. Lu, Z. Wang, J. Wu, and C. Jiang, IQGAP1 promotes pancreatic cancer progression and epithelial-mesenchymal transition (EMT) through Wnt/beta-catenin signaling. Sci Rep 9 (2019) 7539.

[22] S. Piera-Velazquez, and S.A. Jimenez, Endothelial to Mesenchymal Transition: Role in Physiology and in the Pathogenesis of Human Diseases. Physiol Rev 99 (2019) 1281-1324.

[23] X. Sun, Y. Sun, P. Jiang, G. Qi, and X. Chen, Crosstalk between endothelial cell-specific calpain inhibition and the endothelial-mesenchymal transition via the HSP90/Akt signaling pathway. Biomed Pharmacother 124 (2020) 109822.

[24] J.G. Cho, A. Lee, W. Chang, M.S. Lee, and J. Kim, Endothelial to Mesenchymal Transition Represents a Key Link in the Interaction between Inflammation and Endothelial Dysfunction. Front Immunol 9 (2018) 294.

[25] J.R. Lin, Y.J. Zheng, Z.B. Zhang, W.L. Shen, X.D. Li, T. Wei, C.C. Ruan, X.H. Chen, D.L. Zhu, and P.J. Gao, Suppression of Endothelial-to-Mesenchymal Transition by SIRT (Sirtuin) 3 Alleviated the Development of Hypertensive Renal Injury. Hypertension 72 (2018) 350-360.

[26] J. Feng, J. Zhang, A.O. Jackson, X. Zhu, H. Chen, W. Chen, Q. Gui, and K. Yin, Apolipoprotein A1 Inhibits the TGF-beta1-Induced Endothelial-to-Mesenchymal Transition of Human Coronary Artery Endothelial Cells. Cardiology 137 (2017) 179-187.

[27] D. Kloska, A. Kopacz, A. Piechota-Polanczyk, C. Neumayer, I. Huk, J. Dulak, A. Jozkowicz, and A. Grochot-Przeczek, Biliverdin reductase deficiency triggers an endothelial-to-mesenchymal transition in human endothelial cells. Arch Biochem Biophys 678 (2019) 108182.
